# *GmIDD* Is Induced by Short Days in Soybean and May Accelerate Flowering When Overexpressed in *Arabidopsis* via Inhibiting *AGAMOUS-LIKE 18*

**DOI:** 10.3389/fpls.2021.629069

**Published:** 2021-02-26

**Authors:** Xue Yang, Yuntong Zhang, Jinming Shan, Jingzhe Sun, Dongmei Li, Xiaoming Zhang, Wenbin Li, Lin Zhao

**Affiliations:** Key Laboratory of Soybean Biology of Ministry of Education, China (Key Laboratory of Biology and Genetics and Breeding for Soybean in Northeast China), Northeast Agricultural University, Harbin, China

**Keywords:** soybean, photoperiod, flowering, *AGL18*, *GmIDD*

## Abstract

Photoperiod is one of the main climatic factors that determine flowering time and yield. Some members of the INDETERMINATE DOMAIN (IDD) transcription factor family have been reported to be involved in regulation of flowering time in Arabidopsis, maize, and rice. In this study, the domain analysis showed that GmIDD had a typical ID domain and was a member of the soybean IDD transcription factor family. Quantitative real-time PCR analysis showed that *GmIDD* was induced by short day conditions in leaves and regulated by circadian clock. Under long day conditions, transgenic Arabidopsis overexpressing *GmIDD* flowered earlier than wild-type, and *idd* mutants flowered later, while the overexpression of *GmIDD* rescued the late-flowering phenotype of *idd* mutants. Chromatin immunoprecipitation sequencing assays of *GmIDD* binding sites in *GmIDD-overexpression (GmIDD-ox)* Arabidopsis further identified potential direct targets, including a transcription factor, *AGAMOUS-like 18* (*AGL18*). *GmIDD* might inhibit the transcriptional activity of flower repressor *AGL18* by binding to the *TTTTGGTCC* motif of *AGL18* promoter. Furthermore, the results also showed that *GmIDD* overexpression increased the transcription levels of flowering time-related genes *FLOWERING LOCUS T* (*FT*), *SUPPRESSOR OF OVEREXPRESSION OF CONSTANS1* (*SOC1*), *LEAFY* (*LFY*) and *APETALA1* (*AP1*) in Arabidopsis. Taken together, *GmIDD* appeared to inhibit the transcriptional activity of *AGL18* and induced the expression of *FT* gene to promote Arabidopsis flowering.

## Introduction

Soybean [*Glycine max* (L.) Merrill] is a typical short-day (SD) plant, which is particularly sensitive to photoperiod response. SDs can promote flowering, while long-days (LD) can inhibit flower bud growth (Kantolic and Slafer, 2007). Soybean can be regarded as a classical model plant at the beginning of the study of the plant photoperiod pathway due to its photoperiodic response characteristics. The cultivation of soybeans on the earth spans 85 latitudes, with different soybean varieties ranging from Vancouver in the north to New Zealand in the south. However, due to the limitation of latitude, cultivated area of each soybean variety is scarce, resulting in its limited adaptability (Cober and Morrison, 2010). Therefore, flowering time and mature period have become important agronomic traits of soybean, which can influence the yield, quality, and versatility of soybean varieties. By reasonable planting of soybean varieties under different environmental conditions in different regions, the cultivated land resources in this area can be fully utilized, which is of great significance to increase the yield of soybean. Thus, it is a hotspot in the field of reducing the breeding pressure, by exploring new photoperiod genes that control soybean flowering and maturation, and further clarifying the molecular mechanism of these genes involved in soybean photoperiod effect.

INDETERMINATE DOMAIN (IDD) transcription factors are one of the largest and most conserved gene families in the plant kingdom and play an important role in various processes of plant growth and development, such as regulating flower induction (Ali et al., 2019). The IDD transcription factor family has a highly conserved ID domain, which was initially described as consisting of two typical C2H2 and C2HC zinc finger motifs. The functions of some members of the IDD transcription factor family have been identified, particularly in *Arabidopsis*, maize, and rice. In *Arabidopsis*, three IDD family members, MAGPIE/IDD3 (MAG), NUTCRACKER/IDD8 (NUT), and JACKDAW/IDD10 (JKD), and GRAS domain proteins SHR and SCR, have been shown to jointly regulate root development (Levesque et al., 2006; Cui et al., 2007; Welch et al., 2007). SHOOT GRAVITROPISM5/IDD15 (SGR5) was mainly expressed in the endodermis of the inflorescence stem, which was involved in regulating the early gravity perception and starch accumulation of *Arabidopsis* (Long et al., 2015). *AtIDD8* controlled the photoperiod-dependent flowering pathway in *Arabidopsis* by regulating sugar signal transduction (Funck et al., 2012). In maize, *ID1* gene was the first characteristic gene of the IDD transcription factor family and played an important role in regulating the blooming period of corn (Colasanti et al., 1998). *NAKED ENDOSPERM 1* and *2*(*NKD1* and *NKD2*) were two other members of the maize IDD family and involved in seed development (Yi et al., 2015). In rice, *EARLY HEADING DATE 2 (Ehd2)*, a homolog of *ZmID1* in rice, which could up-regulate the expression of the *EARLY HEADING DATE 1 (Ehd1)* gene to promote a FT-like gene *HEADING DATE 3a (Hd3a)* and promote flowering under SDs (Matsubara et al., 2008; Gontarek et al., 2016). *OsId1*, a homologous gene of maize *ID1*, regulates *Ehd1* and its downstream genes, including *Hd3a* and *RICE FLOWERING LOCUS T1* (*RFT1*) to regulate flowering (Park et al., 2008). When *RICE INDETERMINATE 1* (*RID1*) gene was activated to induce phase transition, flowering signals were transduced and regulated through various pathways, and finally integrated with FT-like proteins to induce flowering (Wu et al., 2008). However, the function of IDD transcription factor family members in soybean has not been reported.

MADS-domain proteins form a large and diverse family in plants and play a variety of regulatory roles. Previous reports showed that flowering was determined by the additive effect of multiple MADS-domain flower inhibitors, among which *AGAMOUS-like 15* (*AGL15*) and *AGAMOUS-like 18 (AGL18)* made important contributions (Adamczyk et al., 2007). The double mutants of *AGL18* and *AGL15*, members of the *AGL15-like* branch of the MADS domain regulator, showed early flowering phenotype. It was proposed that *AGL15* and *AGL18* act upstream of the floral integrator FT. The combination of *AGL15* and *AGL18* mutations partially inhibited the photoperiodic pathway defects (Adamczyk et al., 2007).

In the current study, soybean *GmIDD* was identified as a member of IDD transcription factor protein family. Overexpression of *GmIDD* promotes flowering in *Arabidopsis*. Putative target gene *AGL18* and *TTTTGGTCC* motif for DNA binding of *GmIDD* were predicted by ChIP-seq and ChIP-qPCR, and it was preliminarily verified that *GmIDD* promoted flowering by inhibiting *AGL18* activity. Furthermore, the transcription levels of flowering time related genes such as *FT*, *SUPPRESSOR OF OVEREXPRESSION OF CONSTANS1* (*SOC1*), *LEAFY* (*LFY*) and *APETALA1* (*AP1*) were increased by *GmIDD* overexpression in *Arabidopsis*.

## Materials and Methods

### Plant Materials and Growth Conditions

For the diurnal rhythm analysis of *GmIDD* gene, seeds from “DongNong 42,” a photoperiod sensitive soybean variety, were provided by Soybean Research Institute of Northeast Agricultural University (Harbin, China). Seeds of “DongNong 42” were planted in a greenhouse at 25°C with 250 μmol m^–2^ sec^–1^ white light under LDs (16 h/8 h light/dark). When the first trifoliate leaves were expanded, part of the seedlings were transferred to SDs (8 h/16 h light/dark) under the same temperature regime. Seedlings were cultured under LDs and SDs for 30 days (transferred at 15 days) and sampled at 3 h intervals for 24 h, and sampled under continuous light (LL) and dark (DD) conditions for 48 h, and then immediately frozen in liquid nitrogen. Samples of different tissues (including roots, stems, leaves, flowers, pods, and seeds) of plants cultured under LDs and SDs were collected for tissue specific expression analysis of *GmIDD* gene.

In this study, *Arabidopsis thaliana* (Col-0) was used as control and the background plant of genetic transformation. Seeds of the *idd* mutant (SALK_129969c) were obtained from the Arabidopsis Biological Resource Center (ABRC, Columbus, United States). The seeds of *GmIDD-overexpression (GmIDD-ox)*, *idd* mutant, *35S:GmIDD/idd* restoration and wild-type (WT) *Arabidopsis* were surface sterilized with 10% hypochlorite and then planted on MS agar medium. The seeds were placed at 4°C for 72 h and transferred to room temperature (22°C). The 10-day-old seedlings were transplanted into 1:1 vermiculite turbidite soil and cultured under LDs and SDs for flowering phenotype analysis. WT and *GmIDD-ox* transgenic *Arabidopsis* plants were cultured under LDs on MS agar medium for 15 days and sampled for quantitative real-time PCR (qRT-PCR) analysis of flowering time-related genes. These experiments were performed in three biological replicates.

### Plasmid Construction and Generation of Transgenic *Arabidopsis* Plants

For the FLAG and HIS tag construct, we first synthesized the tandem repeats of 3 × FLAG and 6 × Histidine (3F6H) tags with *Not*I at 5′ end and *Xba*I at 3′ end (5′-GCGGCCGCCCTGGAGCTCGGTACCCGGG(*Sma*I)GATCCC AGGATCT**GATTACAAGGATCATGATGGTGATTACAAGG ATCACGACATCGACTACAAGGATGACGATGACAAGCAC CATCATCACCACCATTGA**TCTCTAGA-3′, the sequences encoding 3F6H tag were in bold) (Song et al., 2012). The synthesized products above were cloned into *Not*I*-Xba*I sites of *pENTRY* vector (named *pENTRY-3F6H*) which contained the 3F6H sequence in the C terminus of the cloning site, and the sequences were verified. The full-length coding region of *GmIDD* was amplified from total RNA of “Dongnong 42” using *GmIDD-3F6H-*F and *GmIDD-3F6H-*R primers (Supplementary Table S1). The PCR product was purified and cloned into *pENTRY-3F6H* vector linearized by *Sma*I using In-Fusion cloning system (Clontech, United States) to construct recombinant vector *35S:GmIDD-3F6H-pENTRY*. LR reaction was conducted by *35S:GmIDD-3F6H-pENTRY* and *pB7WG2* to generate *35S:GmIDD-3F6H-pB7WG2* fusion expression vector. The recombinant vector *35S:GmIDD-3F6H -pB7WG2* was introduced into the *Agrobacterium GV3101* which used to transform *Arabidopsis thaliana* (Col-0) and *idd* mutant using the floral dip method with *Agrobacterium tumefaciens* strain GV3101 (Clough and Bent, 1998). Transformants were selected on MS agar medium with 8 mg/L phosphinothricin. T_3_ transgenic seeds of two homozygous lines were selected for further study.

### Subcellular Localization of GmIDD

A cDNA fragment of *GmIDD* was amplified by PCR with *GmIDD-TOPO*-F and *GmIDD-TOPO*-R primers (Supplementary Table S1) from total RNA of “DongNong 42” and cloned into *pENTR/D-TOPO* (Life technologies) and then transferred to the expression vector *pGWB506* through LR reaction to generate the *35S:GFP-GmIDD* fusion vector. The recombinant construct was introduced into *Agrobacterium* GV3101 and subsequently transformed into *N. benthamiana* (Sheikh et al., 2014). After infiltration, the tobacco leaves were grown for 2 days and the GFP signal was detected by fluorescence microscopy.

### ChIP-Seq and ChIP-qPCR

One representative *GmIDD-ox-1* lines were selected for ChIP-seq. *GmIDD-ox-1* transgenic *Arabidopsis* plants were cultured on MS agar medium under LDs for 14 days, and then 1 g seedlings were quick-frozen in liquid nitrogen. With the purpose of minimizing the deviation between different parallel samples under the same treatment, the sample was retrieved from independent three plots of *Arabidopsis* seedlings. After crushing, the seedlings were fixed with 1% (V/V) formaldehyde for 15 min at 4°C. Final concentration of 0.125 M glycine was subsequently added to quench the cross-linking reaction. After nuclei were isolated and lysed, and the chromatin solution was then sonicated to approximately 200–1,000 bp DNA fragments. Immunoprecipitation reactions were performed using Anti-Flag antibody (Monoclonal ANTI-FLAG^®^ M2 antibody produced in mouse, F1804, Sigma-Aldrich). The complex of chromatin antibody was captured with protein G beads (Invitrogen), and DNA was purified using a QIAquick PCR purification kit (QIAGEN). The purified DNA was sent to a sequencing company for library construction and deep sequencing. The ChIP-seq sequencing project was completed on Illumina sequencing platform. Illumina PE library (∼300 bp) was constructed for sequencing and quality control of the obtained sequencing data. Bioinformatics methods were then used to analyze ChIP-seq data. The raw sequence data were aligned to the *Arabidopsis thaliana*(TAIR10)^1^. MACS was used to predict the length of the protein binding sequence through modeling, and the relative abundance of the corresponding peak of the sequence was determined by the length of the sequence and the numbers of pair end reads mapped on the sequence. ChIP-seq was completed by SeqHealth Tech Co., Ltd., Wuhan, China.

ChIP-qPCR was performed using the whole WT and *GmIDD-ox-1* seedlings. The relative enrichment of WT was set to 1. *IPP2* gene (isopentenyl pyrophosphate: dimethyl allyl pyrophosphate isomerase 2, *AT3G02780*) was used as a negative control. DNA samples were analyzed by real-time quantitative PCR using the appropriate DNA primers (Supplementary Table S1) and SuperReal PreMix Plus (TIANGEN, Beijing, China). IgG was used as an antibody control. Data are shown as the mean ± SD of three biological replicates.

### Transient Assay of *AGL18* Promoters Affected by GmIDD in *N. Benthamiana*

To generate *AGL18* promoter driven LUC constructs *proAGL18:LUC*, promoter DNA was amplified from genomic DNA of *Arabidopsis* (Col-0) using *proAGL18:LUC*-F and *proAGL18:LUC*-R primers (Supplementary Table S1). The PCR product was purified and cloned into binary vector *pGreenII-0800-LUC* linearized by *Sma*I using In-Fusion cloning system. The recombinant constructs were introduced into *Agrobacterium* GV3101 and subsequently transformed into *N. benthamiana* (Sheikh et al., 2014). The transient activity of recombinant vectors was assayed using dual-luciferase assay kit (Promega, United States) and Multiscan Spectrum (TECAN Infinite 200 PRO, Männedorf, Switzerland). The construct *35S:GmIDD-3F6H-pB7WG2* and *proAGL18:LUC* were simultaneously transferred into *N. benthamiana* to measure transient assay of the *AGL18* promoters affected by GmIDD protein. A floral repressor *GmRAV* effector construct (*35S:GmRAV-3F6H-pB7WG2*) and *proAGL18:LUC* were simultaneously transferred into *N. benthamiana* as the negative control of this experiment. The recombinant vector *35S::GmRAV-3F6H-pB7WG2* was obtained and introduced into *Agrobacterium* GV3101 by referring to the construction method of *35S::GmIDD-3F6H-pB7WG2* above. Expression vector *pB7WG2* and *proAGL18:LUC* was simultaneously transferred into *N. Benthamiana* as the blank control of this experiment. Three independent experiments were performed and each experiment was repeated three times to obtain reproducible results. The luminescence signal was captured using Amersham Imager 600 (General Electric Company)^2^ after spraying 1 mM luciferin (Heliosense)^3^ on *N. benthamiana* leaves.

### Quantitative RT-PCR Analysis

RNA isolation has been described previously (Zhao et al., 2013). qRT-PCR amplifications were performed using the SuperReal PreMix Plus (TIANGEN, Beijing, China) on Applied Biosystems^TM^ 7500 Fast Dx Real-Time PCR Instrument (ABI). The PCR cycling conditions were as follows: 95°C for 15 min; 40 cycles of 95°C for 10 s, 60°C for 20 s, and 72°C for 30 s. *GmActin4* (GenBank accession number AF049106) and *IPP2* (AT3G02780) were used as endogenous regulatory genes of soybean and *Arabidopsis*, respectively. Three biological replicates and three technical replicates were applied for the whole assays. The primers used in qRT-PCR analyses were shown in Supplementary Table S1.

### Flowering Time Measurements

Flowering time of *Arabidopsis* was determined by scoring the numbers of rosette leaves and the numbers of days from germination to bolting time. At least 20 plants were analyzed each time, and the analysis was repeated for three times.

## Results

### Sequence Analysis of the *GmIDD*

**FIGURE 1:**
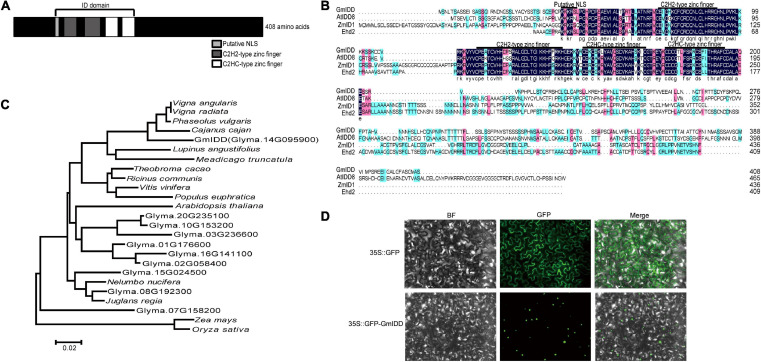
Sequence analysis and subcellular localization of GmIDD. **(A)** Predicted domains of GmIDD paralogs known from Phytozome which contain a nuclear localization signal motif (KKKR) and four zinc finger domains including two types of C2H2 (72–92, 114–142 aa) and two types of C2HC (149–169, 176–195 aa). **(B)** Multiple alignments of the amino acid sequences encoded by *GmIDD* and known IDD family members in *Arabidopsis*, maize, and rice. The nuclear localization signal motif (KKKR) and four zinc finger domains were indicated by the line on the top. Blue, homology 100%; pink, homology ≥ 75%; light blue, homology ≥ 50%. **(C)** Phylogenetic tree analysis was performed on GmIDD and proteins from other species with high similarity in NCBI. All the amino acid sequence information comes from the Phytozome database (accession numbers are listed in Supplementary Table S2). A phylogenetic tree containing 10 soybean proteins with complete ID domain and 15 IDD proteins from other species (*Theobroma cacao*, *Ricinus communis*, *Vitis vinifera*, *Populus euphratica*, *Arabidopsis thaliana*, *Nelumbo nucifera*, *Juglans regia*, *Lupinus angustifolius*, *Cajanus cajan*, *Phaseolus vulgaris*, *Vigna angularis*, *Vigna radiata*, *Zea mays*, *Medicago truncatula*, and *Oryza sativa*. Phylogenetic tree was constructed using the neighbor joining method of MEGA 6.0. **(D)** Subcellular localization of GmIDD protein. The 35S:GFP-GmIDD fusion expression vector was used for agroinfection in *N.benthamiana*. The tobacco leaves infected with 35S:GFP agrobacterium were used as control. After infiltration, the tobacco leaves were grown for 2 days and the GFP signal was detected by fluorescence microscopy. GFP, Green Fluorescent Protein; BF, bright field; Merge, GFP, and bright-field images.

We have identified that both genes such as *GmRAV* and *GmGBP1* induced by SD detected by suppression subtractive hybridization (SSH) functioned in regulating flowering time (Zhao et al., 2008, 2018). In this study, we further detected a zinc finger transcription factor *GmIDD* (*Glyma.14G095900*) gene also induced by SD from SSH library. The *GmIDD* sequence information was obtained from soybean genome database^4^. The cDNA sequence of *GmIDD* is 2,034 bp and contains 1,227 bp open reading frame, which encoded 408 amino acids with predicted molecular mass of 45,129 kDa. The domain analysis showed that GmIDD had a typical ID domain, which consisted of a nuclear localization signal motif (KKKR), four zinc finger domains including two types of C2H2 (72–92 aa and 114–142 aa), and two types of C2HC (149–169 aa and 176–195 aa) (Figure 1A). GmIDD showed high homology with AtIDD8, ZmID1, and Ehd2, which are typical IDD family members in *Arabidopsis*, maize and rice (Figure 1B). Therefore, *GmIDD* is a member of the soybean IDD transcription factor family. A phylogenetic tree containing 10 soybean proteins with complete ID domain and 15 IDD proteins from other species was constructed by MEGA 6.0. Phylogenetic tree analysis showed that GmIDD was located on the same branch with *Vigna angularis* (NC_030643.1), *Cajanus cajan* (KYP72220.1), *Vigna radiata* (XP_017428159.1), *Phaseolus vulgaris* (Phvul.001G036500), *Lupinus angustifolius* (XP_019458210.1), and *Medicago truncatula* (Medtr1g016010.1) (Figure 1C). Soybean IDD proteins Glyma.08G192300 and Glyma.15G024500 were located on the same branch with *Nelumbo nucifera* (xP_010242234.1) and *Juglans regia* (xP_018848593.1) (Figure 1C). Soybean IDD protein Glyma.07G158200 was located on the same branch with *Zea Mays* (GRMZM2G320287) and *Oryza sativa* (LOC_Os02g31890) (Figure 1C). Therefore, the evolution distance between GmIDD and other soybean IDD proteins was relatively far, indicating that soybean IDD protein might have certain differences in function.

### GmIDD Protein Located in Cell Nucleus

The subcellular localization of the GmIDD protein might be crucial for its function. The expression of 35S:GFP-GmIDD fusion protein under the control of the 35S promoter in tobacco mesophyll cells showed that the GmIDD fusion protein was concentrated in the nucleus (Figure 1D), whereas GFP was dispersed throughout the entire tobacco mesophyll cells in the 35S:GFP control. The results clearly showed that GmIDD was a nuclear-localized protein.

### Photoperiod and Circadian Rhythm Regulate the Accumulation of *GmIDD* Transcript

**FIGURE 2:**
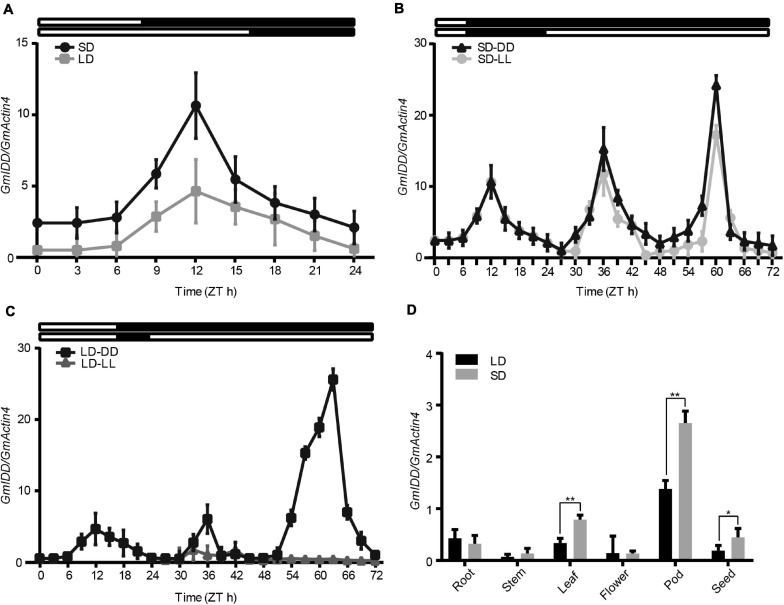
qRT-PCR analyses of soybean *GmIDD* expression patterns. **(A–C)** Soybean “Dongnong 42” plants, which were grown in LDs (16 h/8 h light/dark) for 15 days, were transferred to LDs or SDs (8 h/16 h light/dark) for 15 days for sampling. Trifoliate leaves were sampled at 3 h intervals. **(A)** *GmIDD* diurnal expression under SDs and LDs. **(B)** Expression patterns of *GmIDD* transcripts under constant darkness (DD) and constant light (LL) conditions from SDs. **(C)** Expression patterns of *GmIDD* transcripts under constant darkness (DD) and constant light (LL) conditions from LDs. White and black bars at the top represented light and dark phases, respectively. **(D)** Tissue-specific expression of *GmIDD* at 12 h after dawn under SDs and LDs. All the data were normalized with soybean *GmActin4* gene as internal reference. For each experiment, three technical replicates were conducted. Data shown are mean ± SD of three independent experiments. Significant difference between the expression level of *GmIDD* under SDs and LDs was indicated by asterisk (*P < 0.05 and **P < 0.01, Student’s *t*-test).

The trifoliate leaves of soybean grown for 30 days (transferred at 15 days) were collected every 3 h to show the diurnal expression patterns of *GmIDD* in LDs and SDs by qRT-PCR analysis. *GmIDD* exhibited photoperiod-specific expression patterns under both LDs and SDs. The expression levels of *GmIDD* mRNA under SDs were significantly higher than those under LDs (Figure 2A). To further determine *GmIDD* gene expression patterns, we analyzed *GmIDD* expression under LL and DD conditions after SDs and LDs transfer. *GmIDD* mRNA level maintained a strong rhythm under SDs-DD and SDs-LL, reaching the peak at 12 h after dawn (Figure 2B), but no strong cycling was detected under LDs-LL (Figure 2C). Therefore, *GmIDD* gene was induced by SDs in soybean leaves, and regulated by circadian clock.

### Temporal and Spatial Expression Patterns of *GmIDD* in Soybean

Samples of different tissues (including roots, stems, leaves, flowers, pods, and seeds) of plants cultured under LDs and SDs were collected at 12 h after dawn for tissue specific expression analysis of *GmIDD* gene to determine the expression pattern of *GmIDD* gene during soybean growth and development. The *GmIDD* mRNA was present in all organs examined, which included roots, stems, leaves, flowers, pods, and seeds (Figure 2D). The mRNA abundance of *GmIDD* was the highest in pods among all organs under both SDs and LDs. All the mRNA abundance of *GmIDD* in leaves, stems, pods, and seeds in SDs was higher than that in LDs. However, the mRNA abundance of *GmIDD* in roots in SDs plants was lower than that in LDs. These expression patterns were different from those of *AtIDD8*, which was expressed at a relatively high level in vegetative organs, but at a lower level in pods (Seo et al., 2011). Although *GmIDD* and *AtIDD8* have high amino acid homology and belong to IDD transcription factor family, there may be some differences in biological function.

### Overexpression of *GmIDD* Promotes Flowering in *Arabidopsis*

**FIGURE 3:**
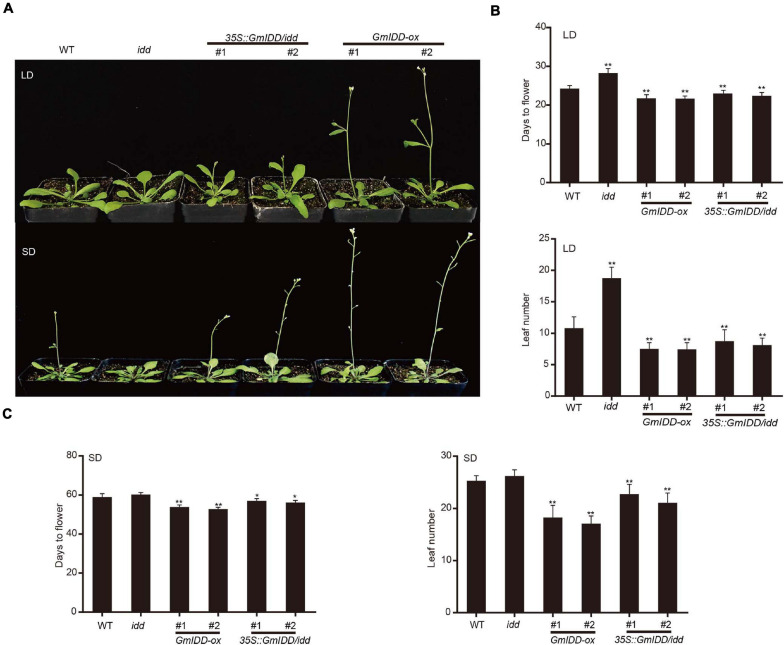
GmIDD conferred early flowering phenotypes in transgenic Arabidopsis. **(A)** Flowering phenotype of *GmIDD* plants under LDs and SDs. *GmIDD-ox*, *idd* mutant, *35S:GmIDD*/*idd* restoration and WT *Arabidopsis* plants were cultured under LDs and SDs. Photographed after the plants have grown in the soil under LDs and SDs for 25 days and 56 days, respectively. **(B)** Days to flowering and average total leaf numbers of *GmIDD-ox*, *idd* mutant, *35S:GmIDD/idd* restoration and WT *Arabidopsis* plants under LDs. **(C)** Days to flowering and average total leaf numbers of *GmIDD-ox*, *idd* mutant, *35S:GmIDD/idd* restoration and WT *Arabidopsis* plants under SDs. Data represent means ± SD of at least 20 seedlings. Asterisks indicate significant differences between *GmIDD-ox*, *idd* mutant, *35S:GmIDD/idd* restoration plants and WT. For each experiment, three technical replicates were conducted. Data shown are mean ± SD of three independent experiments (*P < 0.05 and **P < 0.01, Student’s *t*-test).

In order to verify the function of *GmIDD* in the control of flowering time, the *GmIDD* gene was genetically transformed into WT *Arabidopsis* under the regulation of cauliflower-mosaic virus (CaMV) 35S promoter to obtain *GmIDD-ox* plants. We observed the flowering phenotype of WT *Arabidopsis*, *GmIDD-ox*, *idd* mutant plants under LDs and SDs. Under LDs, the flowering time of *GmIDD-ox* plants were significantly promoted for 3 days, and the *idd* mutants were significantly delayed for 4 days (Figures 3A,B). In addition, the total number of rosette leaves of *GmIDD-ox* plants and *idd* mutants was fewer and more than that of WT plants at bolting, respectively (Figure 3B). Under SDs, the flowering time of *GmIDD-ox* plants were significantly promoted for 5 days, *idd* mutants showed no significant difference (Figures 3A,C). A complementation experiment was conducted on *idd* mutants to further determine the roles of *GmIDD* in promoting flowering time. The phenotype of *idd* mutants was rescued by the expression of a *35S:GmIDD* fusion gene under the control of the 35S promoter. The results indicated that overexpression of *GmIDD* could promote flowering in *Arabidopsis*.

### Genome-Wide Identification of *GmIDD*-Target Genes by ChIP-seq

**FIGURE 4:**
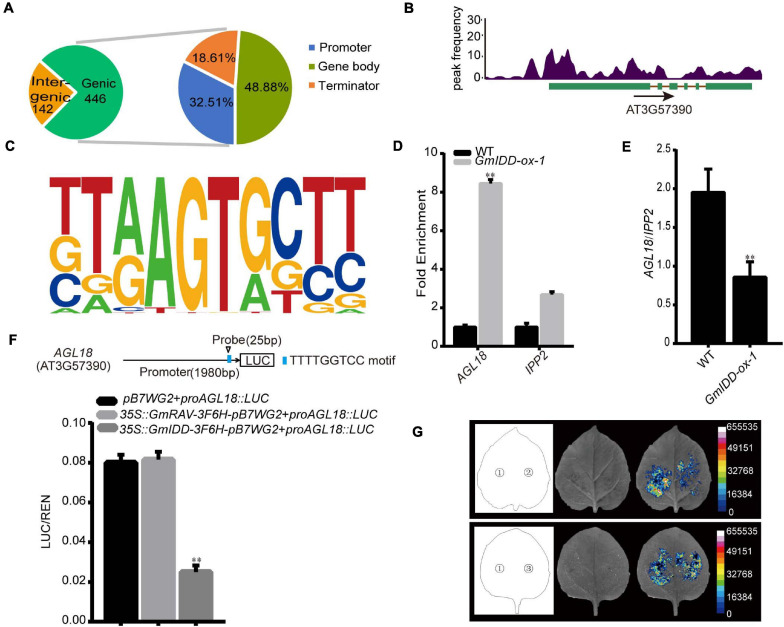
Validation and expression analyses of selected *GmIDD* target genes. **(A)** Distribution of the locations of binding sites relative to target genes. Promoter: a sequence within 2 kb upstream of transcription start site; Terminator: the sequence within 2 kb downstream of the transcription termination site; Gene body: 5′ UTR, CDS, intron, and 3′ UTR. **(B)** Peak graphs showing the ChIP-seq raw reads at the indicated gene loci in Integrative Genomics Viewer. The arrows indicate the directions of transcription, and the green bars indicate the transcripts of gene. **(C)** Motif analysis of *GmIDD*-binding sequences using HOMER software. **(D)** Anti-Flag antibody was used to precipitate chromatin prepared from *GmIDD-ox* transgenic *Arabidopsis* plants. The relative enrichment of the WT fragment was set as 1.0 and those of other fragments were adjusted accordingly. IgG was used as an antibody control. *IPP2* was used as the internal gene control. **(E)** qRT-PCR analysis of the transcript levels of *AGL18* in the 14-day-old WT, and *GmIDD-ox-1* transgenic *Arabidopsis* plants. **(F)** The effect of GmIDD protein on the *AGL18* promoter activity. Relative luciferase activity of co-transfected effector and reporter genes in tobacco leaves was detected in LDs. The activities of firefly LUC were normalized by the activities of 35S:Renilla LUC. Results represent means ± SD of eight independent samples (^∗∗^*P* < 0.01 vs. no effector, Student *t*-test). Upper panel: physical locations of fragments harboring putative motifs are shown in the schematic diagram. **(G)** Luciferase activity of *AGL18* under LDs. 1: *pB7WG2* + *proAGL18:LUC* as the blank control; 2: *35S:GmIDD-3F6H-pB7WG2* + *proAGL18:LUC*; 3: *35S:GmRAV-3F6H-pB7WG2* + *proAGL18:LUC* as the negative control; D-luciferin was used as the substrate of LUC. For each experiment, three technical replicates were conducted. Data shown are mean ± SD of three independent experiments (^∗∗^*P* < 0.01, Student’s *t*-test).

ChIP-seq was performed to identify the DNA binding sites and target genes of *GmIDD* in overexpressed transgenic *Arabidopsis*, and further elucidate the potential mechanism of *GmIDD* in promoting flowering. Among the 588 *GmIDD*-binding sites detected by ChIP-seq, 446 (75.85%) were located in genic regions. These binding sites comprised gene bodies and their flanking regulatory sequences, including 2 kb upstream regions that were assumed to harbor promoter regions and 2 kb downstream regions that were assumed to contain terminator regions. Among the 446 sites in genic regions, 32.51, 48.88, and 18.61% were located in defined promoter regions, gene bodies, and terminator regions, respectively (Figure 4A).

*AGL18 (AT3G57390)* related to flowering time might be a *GmIDD* candidate target. The MADS-domain factor *AGL18* acted redundantly as a repressor of the floral transition in *Arabidopsis* (Adamczyk et al., 2007). *AGL18* acted upstream of the floral integrator *FT*, and a combination of *agl18* and *agl15* mutations partially suppressed defects in the photoperiod pathway. The *GmIDD* binding site in *AGL18* gene regions was located in the promoter region (Figure 4B). According to the consensus sequences at the detected *GmIDD*-binding sites, putative *GmIDD*-binding motifs were predicted using the Hypergeometric Optimization of Motif EnRichment (HOMER) software (Heinz et al., 2010). Based on the prediction, we identified that *GmIDD* combined with target gene *AGL18* motif [(T/G/C/A)(T/G/A)(A/G/C/T)(A/T)(G/T)(T/G)(G/A/T)(C/G/T) (T/C/G)(T/C/G/A)] (*P*-value = 1e-6) (Figure 4C). The previous study reported that *AtIDD8* regulated *SUS4* by binding with the conserved *CTTTTGTCC* motif of its promoter (Seo et al., 2011). Sequence analysis of *AGL18* promoter showed that the *AGL18* promoter contained the *TTTTGGTCC* motif, which was similar to the *CTTTTGTCC* motif of *AtIDD8* combined with *SUS4* promoter.

The identified *GmIDD*-binding sites were further validated by ChIP–qPCR. The 14-day-old *GmIDD-ox-1* transgenic lines were used to perform ChIP-qPCR to verify potential *GmIDD*-binding sites. Anti-Flag antibody was used to precipitate chromatin prepared from *GmIDD-ox-1* transgenic lines. IgG was used as an antibody control. The fragments harboring identified *GmIDD*-binding site in the promoter region of *AGL18* was highly enriched in DNA chromatin-immunoprecipitated (ChIPed) with Anti-Flag in ChIP-qPCR experiments, indicating *GmIDD* bound to the promoter of *AGL18* (Figure 4D).

### GmIDD Inhibits the Transcriptional Function of *AGL18*

It was found that the *AGL18* promoter was bound by *GmIDD* using ChIP-qPCR, and its expression levels was decreased in *GmIDD-ox-1 Arabidopsis* plants using qRT-PCR (Figure 4E). The reporter *proAGL18:LUC* was constructed by *AGL18* promoter containing *GmIDD*-binding site *TTTTGGTCC* motif driving *LUC* reporter gene. When co-infiltrating *Agrobacterium* expressing *35S:GmIDD-3F6H-pB7WG2* effectors together with the *proAGL18:LUC* reporters into tobacco leaves, the activity of LUC was significantly lower than that of blank control of *pB7WG2* and *proAGL18:LUC* co-transformed tobacco leaves (Figures 4F,G), thus demonstrating that *GmIDD* could inhibit the transcriptional activation activities of *AGL18* gene. However, the LUC activity of the floral repressor GmRAV (Zhao et al., 2008) and *proAGL18:LUC* co-transformed tobacco leaves showed no significant difference compared with the blank control. Together, our results suggested that GmIDD protein significantly repressed the expression of *AGL18* by directly binding to its promoter.

### *GmIDD* Affects the Expression of Flowering Time-Related Genes

**FIGURE 5:**
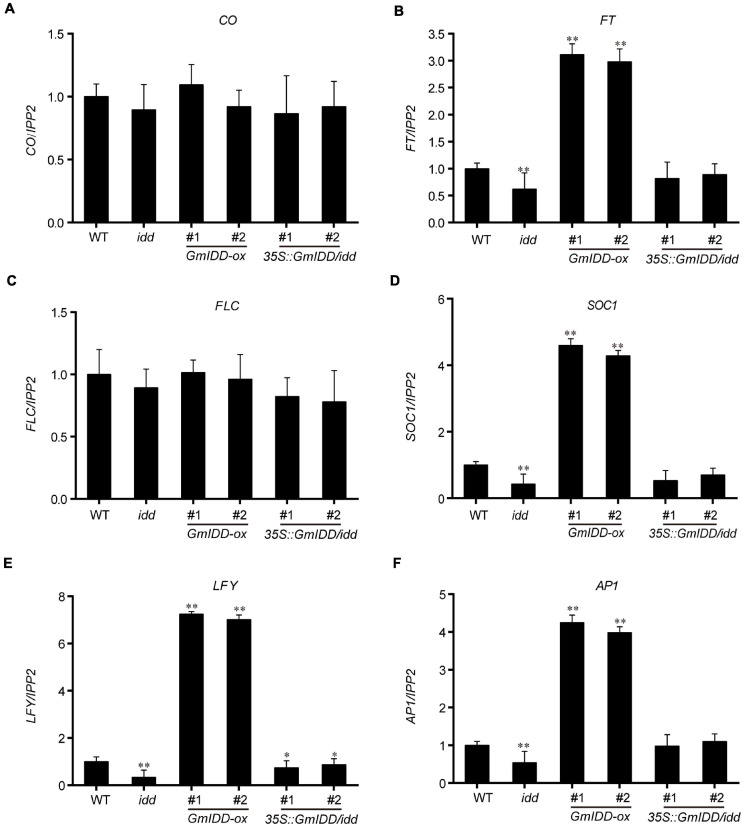
Expression levels of flowering time-related genes in *GmIDD-ox*, *idd* mutants, *35S:GmIDD/idd* restoration and WT *Arabidopsis* plants were investigated by qRT-PCR. WT and *GmIDD*-transgenic plants were cultured in LDs for 15 days, and sampled 12 h after dawn for qRT-PCR analysis the expression levels of flowering time-related genes to determine the mechanism of *GmIDD* regulating flowering time. **(A–F)** Relative expression of the *CO*, *FT*, *FLC*, *SOC1*, *LFY*, and *AP1*, respectively. For each experiment, three technical replicates were conducted. Data shown are mean ± SD of three independent experiments, the significant differences between *GmIDD*-transgenic and WT plants are indicated by asterisks (^∗^*P* < 0.05 and ^∗∗^*P* < 0.01, Student’s *t*-test).

Overexpression of *GmIDD* shortened the flowering time of *Arabidopsis*. The expression levels of flowering time-related genes (including *CO*, *FT*, *FLC*, *SOC1*, *LFY*, and *AP1*) in the 15-day-old *GmIDD*-transgenic seedlings were further investigated to determine the mechanism of *GmIDD* regulating flowering time. The results showed that the mRNA levels of *FT*, floral homeotic gene *AP1*, floral meristem identity gene *LFY* and floral integrator gene *SOC1* were obviously increased in the *GmIDD-ox* transgenic plants compared to WT plants, but they were significantly decreased in *idd* mutants. In *35 S:GmIDD/idd* complementary lines, the down-regulated trend of *FT*, *AP1*, *LFY*, and *SOC1* genes were rescued (Figures 5B,D–F). The expression of *CO*, *FLC* and the other genes functioning in the autonomous pathway showed no significant difference compared to WT (Figures 5A,C). *FLC* is a floral repressor of the vernalization and autonomous pathway and *GmIDD* might not affect flowering time through the autonomous pathway. Taken together, *GmIDD* inhibited the transcriptional activities of *AGL18* gene, and induced the expression of *FT* gene to promote flowering in *Arabidopsis*.

## Discussion

INDETERMINATE DOMAIN (IDD) is a plant-specific subfamily of C2H2 zinc finger transcription factors (Colasanti et al., 2006; Seo et al., 2011). IDD transcription factor family shares a highly conserved N-terminal domain (ID domain) consisting of two C2H2, two C2HC zinc finger domains and a nuclear localization signal (Englbrecht et al., 2004). The functions of some members of the IDD transcription factor family have been identified, particularly in *Arabidopsis*, maize, and rice. However, the function of IDD transcription factor family members in soybean has not been reported. This study analyzed the structural domains of GmIDD protein and found that GmIDD consisted of four zinc finger domains: two types of C2H2 (72-92 aa and 114–142 aa) and two types of C2HC (149–169 aa, and 176–195 aa) (Figure 1A). According to the subcellular localization of GmIDD in tobacco leaf cells, the GmIDD-GFP fusion protein was specifically localized on the nucleus of tobacco leaf cells, which indicated that GmIDD was a predicted nuclear transcriptional regulator and performed its biological functions in the plant nucleus (Figure 1D).

Soybean is a typical SD plant that is particularly sensitive to photoperiod response. Photoperiod is one of the main climatic factors that determine soybean floral development (Câmara et al., 1997). Maize *ID1* gene expression and protein levels were basically not affected by the change of day length and did not follow the circadian rhythm pattern, indicating that the expression of *ID1* was not controlled by photoperiod (Coneva et al., 2007). Similar to *ID1*, rice *Ehd2* was not regulated by daylength, but *Ehd2* was involved in photoperiod inducing pathway (Matsubara et al., 2008). The expression of *Arabidopsis AtIDD8* gene level was basically not affected by the change of day length, but might be involved in photoperiod inducing pathway to promote *Arabidopsis* flowering (Seo et al., 2011). Expression pattern analysis of *GmIDD* showed that expression level of *GmIDD* was induced by SDs and maintained a stable circadian rhythm under SDs (Figures 2A,B). *GmIDD-ox Arabidopsis* plants showed early flowering phenotype under LDs and SDs. The *idd* mutants showed late flowering phenotype under LDs but not SDs. The phenotypes of *35S:GmIDD/idd* complementary lines were rescued compared with *idd* mutants (Figure 3A). Therefore, *GmIDD* expression was regulated by daylength and might be involved in promoting of *Arabidopsis* flowering time. The different photoperiodic responses of *Ehd2* and maize *ID1* could be due to functional differentiation of downstream genes (Matsubara et al., 2008). This may also be the reason why the response of *GmIDD* to the change of day length was different from that of *Ehd2* and *ID1*, but this speculation has not been confirmed. Both *ID1* and *Ehd2* were specifically expressed in developing leaves (Kozaki et al., 2004; Wong and Colasanti, 2007). *GmIDD* mRNA in soybean tissues accumulated most abundantly in pods (Figure 2D), but was present at very low levels in leaves, patterns that are different from *ID1* to *Ehd2*. *ID1* regulated a leaf-derived floral inductive signal that might be unique to monocots (Colasanti et al., 2006). Soybean belongs to dicotyledonous. The mechanism of *GmIDD* regulating flowering in soybean might be different from *ID1* and needs to be clarified. *Arabidopsis AtIDD8* transcript levels were relatively high in vegetative organs, but at a lower level in pods (Seo et al., 2011). This suggests that *GmIDD* and *AtIDD8* may be functionally distinct in some aspects, although both promote *Arabidopsis* flowering.

Maize ID1 is a founding member of the plant-specific zinc finger protein family, which consists of highly conserved amino acid sequences in the ID domain (Colasanti et al., 1998). Previous studies had found that *ID1*, as a regulator of gene expression, bound to specific 11 bp DNA consensus motif *5-TTTGTCG/CT/CT/aT/aT-3 in vitro*. It was also proposed that all proteins with ID domains had unique DNA binding characteristics and could recognize the same DNA target sequence (Kozaki et al., 2004). Furthermore, it also showed that *AtIDD8* regulated *SUS4* by binding with the conserved *CTTTTGTCC* motif of its promoter (Seo et al., 2011). To identify possible targets of GmIDD protein, the target genes of *GmIDD* binding were predicted by ChIP-seq analysis using *GmIDD-ox* and WT *Arabidopsis* as materials. A flowering-related gene *AGL18* was finally identified by screening the genes that were bound by *GmIDD* to the promoter region. *AGL18* is a member of the MADS-box gene family. MADS-box proteins influenced root growth of *Arabidopsis* (Zhang and Forde, 1998), and also influenced development of ovules, fruits, and seed coat (Nesi et al., 2002; Pinyopich et al., 2003). In addition, *AGL18* was a floral repressor in *Arabidopsis* and located upstream of the *FT* (Adamczyk et al., 2007). *AGL18* promoter region has a continuous *TTTTGGTCC* motif similar to the reported *AtIDD8* and *SUS4* binding motif *CTTTTGTCC*. This difference might be due to differences between species. *AGL18* was highly enriched in chromatin of *GmIDD-ox* transgenic lines precipitated by anti-marker antibodies (Figure 4D). The presence of GmIDD protein reduced the transcriptional activity of *AGL18* (Figures 4F,G). qRT-PCR analysis of *GmIDD-ox* plants showed that the expression level of *AGL18* decreased (Figure 4E) while mRNA levels of *FT*, *AP1*, *LFY*, and *SOC1* increased significantly (Figures 5B,D–F).

## Conclusion

In conclusion, the results of this study indicated that GmIDD molecular regulatory mechanism played a key role in the regulation of flowering in transgenic *Arabidopsis*. *GmIDD* binding with *TTTTGGTCC* motif of target gene *AGL18* promoter inhibited the expression of *AGL18*, thus weakened the inhibition of *AGL18* on *FT* and finally promoted flowering of *Arabidopsis*. The *CO*/*FT* module had been demonstrated in *Arabidopsis* flowering regulation. In this module, CONSTANS (CO) transcription factor promoted the transcription of *FT*. FT protein interacted with FD-encoded transcription factor to activate downstream floral organ genes and thus induced flowering (Kardailsky et al., 1999; Valverde et al., 2004; Abe et al., 2005). It has been proposed that the common *CO*/*FT* induction pathway was conserved during photoperiodic regulation of flowering in *Arabidopsis* and rice (Matsubara et al., 2008). In rice, *Ehd2* was located in the upstream of *HEADING DATE1 (Hd1)* (a *CO* ortholog in rice) and promoted the expression of *FT* by up-regulating the expression of *Hd1*, thus promoting flowering. However, the overexpression of *GmIDD* promoted the expression of *FT* while the transcription level of *CO* remained unchanged (Figures 5A,B). This phenomenon showed that the functions of members of IDD transcription factor family had some similarities but also some differences. Therefore, the molecular regulation mechanism of *GmIDD* in soybean needs to be further investigated.

## Data Availability Statement

The datasets presented in this study can be found in online repositories. The names of the repository/repositories and accession number(s) can be found below: NCBI-SRA database under the BioProject no. PRJNA681294 and accession Nos. SRR13165421 and SRR13165420 for the ChIP-seq data.

## Author Contributions

XY performed the gene cloning and transformation and ChIP assays. YZ and JSh performed the phenotype observations and measurements. JSu, DL, and XZ performed the dual-luciferase assay and data analysis. XY, LZ, and WL wrote the manuscript. All authors contributed to the article and approved the submitted version.

## Conflict of Interest

The authors declare that the research was conducted in the absence of any commercial or financial relationships that could be construed as a potential conflict of interest.

## Footnotes

1 https://www.arabidopsis.org/

2 https://www.ge.com/

3 http://www.heliosense.com/

4 https://phytozome.jgi.doe.gov/
