# Supplementary material for: GmIDD Is Induced by Short Days in Soybean and May Accelerate Flowering When Overexpressed in Arabidopsis via Inhibiting AGAMOUS-LIKE 18
Source: Front Plant Sci. 2021 Feb 26;12:629069. doi: 10.3389/fpls.2021.629069 (PMC8029582; doi:10.3389/fpls.2021.629069)
Supplement: Supplementary file 1 [file Data_Sheet_1.pdf]

Table S1. The specific sequences of the primers.

Primer	Sequence (5’-3’)
GmIDD-3F6H-F	TGGAGCTCGGTACCCATGTCCAATTTGACGTCTGCAT
GmIDD-3F6H-R	GATCCTGGGATCCCCGAAGATGCCATGCCACTAGCAAAC
GmIDD-TOPO-F	CACCATGTCCAATTTGACGTCTGCAT
GmIDD-TOPO-R	TCAAGATGCCATGCCACTAGCAAAC
GmRAV-3F6H-F	TGGAGCTCGGTACCCATGGATGGAGGCTGTGTCACAG
GmRAV-3F6H-R	GATCCTGGGATCCCCGACAAAGCTCCAATTACTTTTAACTTC
qAtAGL18- ChIP-F	GGGAGGCGATACACGAAATAG
qAtAGL18- ChIP-R	GGAGGACTAGTAGAGGAAGGGGTT
proAGL18::LUC-F	GAATTCCTGCAGCCCTTTGGAGATGTAAACATTTTGTACAG
proAGL18::LUC-R	ACTAGTGGATCCCCCGGAGGACTAGTAGAGGAAGGGGTT
qAtAGL18 -F	TCTAAGAGACGAAACGGTTTGA
qAtAGL18 -R	CACAGCTTCATTTCCATGTGAA
qGmActin4 -F	GTGTCAGCCATACTGTCCCCATTT
qGmActin4-R	GTTTCAAGCTCTTGCTCGTAATCA
qGmIDD-F	CTCCAACAGTGGTAGAAGAAGCAA
qGmIDD-R	AGGGAAGGAACTCAAAGAAGAAAG
qAtIPP2-F	TGAGCTTGGTATTGTAGCTGAA
qAtIPP2-R	GACAGTTTCAAACCTTCCTCAC
qAtCO-F	AAGGTGATAAGGATGCCAAGGAG
qAtCO-R	GGAGCCATATTTGATATTGAACTGA
qAtFT-F	TGGTGGAGAAGACCTCAGGAAC
qAtFT-R	TGCCAAGCTGTCGAAACAATAT
qAtFLC-F	GCTCTTCTCGTCGTCTCC
qAtFLC-R	GTTCGGTCTTCTTGGCTC
qAtSOC1-F	TCAGAACTTGGGCTACTC
qAtSOC1-R	TTCTCGTCGTCTCCGCCTCC
qAtLFY-F	TGTGAACATCGCTTGTCGTC
qAtLFY-R	TAATACCGCCAACTAAAGCC
qAtAP1-F	TAAGCACATCCGCACTAG
qAtAP1-R	TTCTTGATACAGACCACCC

Table S2. Plant IDD proteins’ information.

Species name	Accession number
Glycine max	Glyma.14G095900
Glycine max	Glyma.03G236600
Glycine max	Glyma.16G141100
Glycine max	Glyma.02G058400
Glycine max	Glyma.01G176600
Glycine max	Glyma.20G235100

Glycine max	Glyma.10G153200
Glycine max	Glyma.07G158200
Glycine max	Glyma.08G192300
Glycine max	Glyma.15G024500
Theobroma cacao	Thecc1EG037300
Ricinus communis	29794.t000003
Vitis vinifera	GSVIVG01035391001
Populus euphratica	XP_011014257.1
Arabidopsis thaliana	AT5G44160.1
Nelumbo nucifera	XP_010242234.1
Juglans regia	XP_018848593.1
Lupinus angustifolius	XP_019458210.1
Cajanus cajan	KYP72220.1
Phaseolus vulgaris	Phvul.001G036500
Vigna angularis	NC_030643.1
Vigna radiata	XP_017428159.1
Zea mays	GRMZM2G320287
Oryza sativa	LOC_Os02g31890
Medicago truncatula	Medtr1g016010.1

Supplemental Data Set 1. List of GmIDD binding sites in the genome.

This file lists the genomic sites bound by GmIDD in the ChIP-seq experiments.

Gene	Chromosome	Start	End	Strand	Distance to TSS	Region	Putative Function
AT1G02130	Chr01	401308	401542	-	494	P	ARABIDOPSIS RAS 5
AT1G11710	Chr01	3949293	3949630	+	0	G	Pentatricopeptide repeat (PPR) superfamily protein
AT1G72440	Chr01	27274326	27274560	-	-508	P	CCAAT-binding factor
AT1G72700	Chr01	27367258	27367507	+	0	G	ATPase E1-E2 type family protein / haloacid dehalogenase-like hydrolase family protein
AT1G72700	Chr01	27368983	27369217	+	0	G	ATPase E1-E2 type family protein / haloacid dehalogenase-like hydrolase family protein
AT1G73540	Chr01	27646118	27646518	-	425	P	nudix hydrolase homolog 21
AT1G75400	Chr01	28297587	28297821	+	0	G	RING/U-box superfamily protein
AT1G76270	Chr01	28617990	28618255	-	0	G	O-fucosyltransferase family protein
AT1G77690	Chr01	29203164	29203721	-	-24	P	like AUX1 3
AT1G78790	Chr01	29624919	29625153	-	42	P	centromere protein X
AT1G79990	Chr01	30089146	30089380	+	0	G	coatomer subunit beta-2

AT1G80810	Chr01	30365551	30365785	+	0	G	Tudor/PWWP/MBT superfamily protein
AT1G13230	Chr01	4519806	4520040	+	-636	P	Leucine-rich repeat (LRR) family protein
AT1G13270	Chr01	4545646	4545880	+	0	G	methionine aminopeptidase 1B
AT1G13450	Chr01	4614822	4615071	-	313	P	Homeodomain-like superfamily protein
AT1G14320	Chr01	4888501	4888740	+	406	P	Ribosomal protein L16p/L10e family protein
AT1G15110	Chr01	5199107	5199486	+	23	P	phosphatidyl serine synthase family protein
AT1G16630	Chr01	5682655	5682961	-	0	G	transmembrane protein
AT1G16705	Chr01	5712315	5712650	+	394	T	p300/CBP acetyltransferase-related protein-like protein
AT1G17880	Chr01	6153740	6153974	-	125	T	basic transcription factor 3
AT1G03630	Chr01	908783	909112	+	0	G	protochlorophyllide oxidoreductase C
AT1G18485	Chr01	6363615	6364072	+	801	T	Pentatricopeptide repeat (PPR) superfamily protein
AT1G19290	Chr01	6666884	6667197	+	0	G	Pentatricopeptide repeat (PPR) superfamily protein
AT1G19720	Chr01	6821934	6822200	-	644	T	Pentatricopeptide repeat (PPR-like) superfamily protein
AT1G20880	Chr01	7264802	7265384	-	352	T	RNA-binding (RRM/RBD/RNP motifs) family protein
AT1G21245	Chr01	7436816	7437050	+	91	P	Protein kinase superfamily protein
AT1G21850	Chr01	7672017	7672251	-	0	G	SKU5 similar 8
AT1G21950	Chr01	7722092	7722344	-	1736	T	transmembrane protein
AT1G21980	Chr01	7735529	7735776	+	0	G	phosphatidylinositol-4-phosphat e 5-kinase 1
AT1G22940	Chr01	8121520	8121754	+	-702	P	thiamin biosynthesis protein
AT1G04530	Chr01	1234983	1235217	-	0	G	Tetratricopeptide repeat (TPR)-like superfamily protein
AT1G23220	Chr01	8243184	8243418	+	0	G	Dynein light chain type 1 family protein
AT1G23360	Chr01	8296163	8296452	-	616	T	S-adenosyl-L-methionine-depen dent methyltransferases superfamily protein
AT1G24735	Chr01	8759701	8759935	+	1241	T	S-adenosyl-L-methionine-depen dent methyltransferases superfamily protein
AT1G24800	Chr01	8768885	8769153	+	-492	P	F-box family protein

AT1G25540	Chr01	8972807	8973041	-	0	G	phytochrome and flowering time regulatory protein (PFT1)
AT1G24400	Chr01	8653336	8653570	-	0	G	lysine histidine transporter 2
AT1G27770	Chr01	9674238	9674472	-	0	G	autoinhibited Ca2+-ATPase 1
AT1G28265	Chr01	9878962	9879229	+	60	P	hypothetical protein
AT1G29670	Chr01	10375880	10376129	+	0	G	GDSL-like Lipase/Acylhydrolase superfamily protein
AT1G30060	Chr01	10544851	10545167	-	897	T	COP1-interacting protein-like protein
AT1G05320	Chr01	1554815	1555049	+	0	G	myosin heavy chain, embryonic smooth protein
AT1G30400	Chr01	10728309	10728628	+	0	G	multidrug resistance-associated protein 1
AT1G31390	Chr01	11244977	11245211	-	-702	P	TRAF-like family protein
AT1G31430	Chr01	11254918	11255275	-	641	T	Pentatricopeptide repeat (PPR-like) superfamily protein
AT1G32361	Chr01	11675448	11675782	+	84	P	RING/U-box superfamily protein
AT1G32415	Chr01	11696099	11696333	+	620	T	pentatricopeptide (PPR) repeat-containing protein
AT1G32530	Chr01	11761857	11762153	-	0	G	RING/U-box superfamily protein
AT1G33060	Chr01	11975587	11975898	-	0	G	NAC 014
AT1G34010	Chr01	12360283	12360517	-	809	P	hypothetical protein
AT1G34130	Chr01	12430520	12430762	+	795	T	staurosporin and temperature sensitive 3-like b
AT1G34575	Chr01	12658520	12658817	-	88	P	FAD-binding Berberine family protein
AT1G05350	Chr01	1563096	1563334	-	0	G	NAD(P)-binding Rossmann-fold superfamily protein
AT1G39070	Chr01	14736140	14736447	+	0	G	hypothetical protein
AT1G40104	Chr01	15082104	15085897	+	0	G	hypothetical protein
AT1G42990	Chr01	16136212	16136491	-	1112	T	basic region/leucine zipper motif 60
AT1G05670	Chr01	1701308	1701546	-	-362	P	Pentatricopeptide repeat (PPR-like) superfamily protein
AT1G45223	Chr01	17141185	17141419	+	0	G	ECA1 gametogenesis family protein (DUF784)
AT1G47260	Chr01	17322779	17323430	-	0	G	gamma carbonic anhydrase 2
AT1G49450	Chr01	18304859	18305093	+	-469	P	Transducin/WD40 repeat-like superfamily protein
AT1G50170	Chr01	18582517	18582867	+	0	G	sirohydrochlorin ferrochelatase

							B
AT1G50180	Chr01	18586400	18586634	+	0	G	NB-ARC domain-containing disease resistance protein
AT1G50290	Chr01	18629911	18630145	-	0	G	hypothetical protein
AT1G52150	Chr01	19413942	19414262	-	0	G	Homeobox-leucine zipper family protein / lipid-binding START domain-containing protein
AT1G52615	Chr01	19601291	19601525	-	937	P	ncRNA
AT1G52620	Chr01	19604352	19604730	+	745	T	Pentatricopeptide repeat (PPR) superfamily protein
AT1G06520	Chr01	1995806	1996143	-	0	G	glycerol-3-phosphate acyltransferase 1
AT1G53350	Chr01	19905244	19905478	+	0	G	Disease resistance protein (CC-NBS-LRR class) family
AT1G55080	Chr01	20553946	20554180	-	191	P	mediator of RNA polymerase II transcription subunit-like protein
AT1G55480	Chr01	20714644	20714953	+	1299	T	protein containing PDZ domain, a K-box domain, and a TPR region
AT1G55915	Chr01	20906998	20907453	+	-252	P	zinc ion binding protein
AT1G56085	Chr01	20977153	20977429	+	776	P	Cyclophilin
AT1G56345	Chr01	21093631	21094029	-	631	P	Pseudouridine synthase family protein
AT1G56690	Chr01	21254225	21254682	+	0	G	Pentatricopeptide repeat (PPR) superfamily protein
AT1G57980	Chr01	21439938	21440172	-	0	G	Nucleotide-sugar transporter family protein
AT1G11270	Chr01	3781911	3782319	-	0	G	F-box and associated interaction domains-containing protein
AT1G59124	Chr01	21817472	21818797	+	0	G	Disease resistance protein (CC-NBS-LRR class) family
AT1G60570	Chr01	22312634	22312879	+	0	G	Galactose oxidase/kelch repeat superfamily protein
AT1G08377	Chr01	22612787	22613021	+	646	T	NA
AT1G61970	Chr01	22904687	22904921	+	318	T	Mitochondrial transcription termination factor family protein
AT1G65346	Chr01	24274579	24275053	-	-778	P	hypothetical protein
AT1G65346	Chr01	24275231	24275639	-	0	G	hypothetical protein
AT1G65470	Chr01	24323451	24323685	-	0	G	chromatin assembly factor-1 (FASCIATA1) (FAS1)
AT1G65541	Chr01	24365446	24365905	+	-391	P	hypothetical protein

AT1G66320	Chr01	24728194	24728428	-	0	G	F-box/RNI-like superfamily protein
AT1G66960	Chr01	24991777	24992011	-	-128	P	Terpenoid cyclases family protein
AT1G67110	Chr01	25072156	25072390	-	0	G	cytochrome P450, family 735, subfamily A, polypeptide 2
AT1G68060	Chr01	25515494	25515766	-	0	G	microtubule-associated proteins 70-1
AT1G68920	Chr01	25915446	25915944	+	0	G	basic helix-loop-helix (bHLH) DNA-binding superfamily protein
AT1G69280	Chr01	26048350	26048671	-	-320	P	hypothetical protein
AT1G70530	Chr01	26590511	26590778	-	0	G	cysteine-rich RLK (RECEPTOR-like protein kinase) 3
AT1G71235	Chr01	26854612	26854846	+	170	P	hypothetical protein
AT1G71420	Chr01	26919088	26919322	-	0	G	Tetratricopeptide repeat (TPR)-like superfamily protein
AT1G72050	Chr01	27115943	27116191	+	0	G	transcription factor IIIA
AT2G01008	Chr02	1157	2097	+	0	G	maternal effect embryo arrest protein
AT2G01008	Chr02	2296	3776	+	0	G	maternal effect embryo arrest protein
AT2G01021	Chr02	4175	7526	+	-721	P	hypothetical protein
AT2G01023	Chr02	8384	10321	-	415	T	hypothetical protein
AT2G04485	Chr02	947279	947576	-	0	G	NA
AT2G03290	Chr02	999324	999558	+	35	P	emp24/gp25L/p24 family/GOLD family protein
AT2G03480	Chr02	1051482	1051818	+	0	G	QUASIMODO2 LIKE 2
AT2G05590	Chr02	2067912	2068146	+	0	G	TLD-domain containing nucleolar protein
AT2G06025	Chr02	2347881	2348115	+	-61	P	Acyl-CoA N-acyltransferases (NAT) superfamily protein
AT2G07771	Chr02	3239782	3240214	-	465	T	Cytochrome C assembly protein
AT2G07671	Chr02	3251741	3252380	-	281	P	ATP synthase subunit C family protein
AT2G07674	Chr02	3268247	3268588	+	-515	P	transmembrane protein
AT2G07681	Chr02	3290206	3290664	+	0	G	Cytochrome C assembly protein
AT2G07772	Chr02	3301653	3301896	-	30	P	hypothetical protein
AT2G07698	Chr02	3362623	3363042	+	0	G	ATPase, F1 complex, alpha subunit protein
AT2G07701	Chr02	3370325	3371851	+	-744	P	hypothetical protein
AT2G07638	Chr02	3376734	3377371	+	-111	P	hypothetical protein
AT2G07708	Chr02	3387537	3387818	+	300	T	hypothetical protein

AT2G07641	Chr02	3390729	3391104	-	-190	P	hypothetical protein
AT2G07644	Chr02	3405404	3405640	+	26	P	hypothetical protein
AT2G07646	Chr02	3408881	3409436	+	639	P	hypothetical protein
AT2G07722	Chr02	3437567	3438418	+	-750	P	transmembrane protein
AT2G07722	Chr02	3438610	3438871	+	-2	P	transmembrane protein
AT2G07652	Chr02	3442683	3443024	-	-1539	T	hypothetical protein
AT2G07725	Chr02	3447340	3448403	+	473	P	Ribosomal L5P family protein
AT2G07727	Chr02	3450946	3452094	+	0	G	cytochrome b
AT2G07825	Chr02	3474335	3474582	-	206	P	hypothetical protein
AT2G07658	Chr02	3479431	3480102	-	-570	P	hypothetical protein
AT2G07659	Chr02	3480527	3480883	+	-331	P	transmembrane protein
AT2G07659	Chr02	3481041	3481429	+	0	G	transmembrane protein
AT2G07806	Chr02	3486031	3486451	+	1411	T	hypothetical protein
AT2G07739	Chr02	3495779	3496065	-	0	G	Ycf1 protein
AT2G07741	Chr02	3502833	3503389	+	792	P	ATPase, F0 complex, subunit A protein
AT2G08986	Chr02	3614904	3622090	+	439	T	hypothetical protein
AT2G08986	Chr02	3622369	3622820	+	0	G	hypothetical protein
AT2G08986	Chr02	3623085	3623960	+	0	G	hypothetical protein
AT2G08986	Chr02	3624223	3624821	+	0	G	hypothetical protein
AT2G08986	Chr02	3625090	3626499	+	0	G	hypothetical protein
AT2G13100	Chr02	5390556	5390794	+	0	G	Major facilitator superfamily protein
AT2G14830	Chr02	6365239	6365599	+	0	G	Ist1p
AT2G15050	Chr02	6518827	6519067	+	0	G	lipid transfer protein
AT2G16586	Chr02	7190937	7191366	+	214	P	transmembrane protein
AT2G17390	Chr02	7556226	7556460	+	0	G	ankyrin repeat-containing 2B
AT2G18730	Chr02	8119511	8119745	+	0	G	diacylglycerol kinase 3
AT2G21470	Chr02	9198652	9198913	+	0	G	SUMO-activating enzyme 2
AT2G21770	Chr02	9284282	9284609	+	54	P	cellulose synthase A9
AT2G22100	Chr02	9392354	9392970	-	0	G	RNA-binding (RRM/RBD/RNP motifs) family protein
AT2G22510	Chr02	9569462	9569760	-	0	G	hydroxyproline-rich glycoprotein family protein
AT2G22610	Chr02	9600182	9600416	+	0	G	Di-glucose binding protein with Kinesin motor domain-containing protein
AT2G25060	Chr02	10662200	10662434	+	127	T	early nodulin-like protein 14
AT2G25320	Chr02	10786874	10787266	-	0	G	TRAF-like family protein
AT2G25740	Chr02	10980499	10980791	+	523	T	ATP-dependent protease La (LON) domain protein
AT2G25930	Chr02	11059076	11059656	+	422	T	hydroxyproline-rich glycoprotein family protein
AT2G08175	Chr02	11408971	11409268	-	189	P	NA

AT2G27650	Chr02	11798958	11799227	-	0	G	Ubiquitin carboxyl-terminal hydrolase-related protein
AT2G29020	Chr02	12470536	12470884	+	0	G	Rab5-interacting family protein
AT2G29930	Chr02	12757483	12757765	-	0	G	F-box/RNI-like superfamily protein
AT2G29950	Chr02	12768215	12768499	+	772	T	ELF4-like 1
AT2G30110	Chr02	12856171	12856405	-	0	G	ubiquitin-activating enzyme 1
AT2G30590	Chr02	13033505	13033739	+	0	G	WRKY DNA-binding protein 21
AT2G31840	Chr02	13538949	13539200	-	0	G	Thioredoxin superfamily protein
AT2G08500	Chr02	13589475	13589709	-	-679	P	NA
AT2G34570	Chr02	14563880	14564388	-	0	G	PIN domain-like family protein
AT2G08670	Chr02	14634215	14634449	+	1184	T	NA
AT2G34740	Chr02	14656465	14656699	+	0	G	protein phosphatase 2C family protein
AT2G35060	Chr02	14776955	14777239	-	0	G	K+ uptake permease 11
AT2G35170	Chr02	14828396	14828666	+	0	G	Histone H3 K4-specific methyltransferase SET7/9 family protein
AT2G35637	Chr02	14984332	14984566	-	-110	P	ncRNA
AT2G36355	Chr02	15242432	15242666	+	-54	P	RAB6-interacting golgin (DUF662)
AT2G37070	Chr02	15578758	15579063	+	495	T	TRIO/F-actin-binding protein
AT2G37400	Chr02	15695965	15696199	-	1375	T	Tetratricopeptide repeat (TPR)-like superfamily protein
AT2G39230	Chr02	16382432	16382848	+	0	G	LATERAL ORGAN JUNCTION
AT2G39355	Chr02	16437654	16437957	+	1301	T	F-box/associated interaction domain protein
AT2G40000	Chr02	16701372	16701912	-	0	G	ortholog of sugar beet HS1 PRO-1 2
AT2G40730	Chr02	16992137	16992371	-	0	G	kinase family with ARM repeat domain-containing protein
AT2G42650	Chr02	17766233	17766467	+	1172	T	Ribosomal protein L1p/L10e family
AT2G43430	Chr02	18037589	18037823	-	0	G	glyoxalase 2-1
AT2G44350	Chr02	18316510	18316744	+	0	G	Citrate synthase family protein
AT2G45330	Chr02	18686628	18686925	+	464	P	RNA 2'-phosphotransferase, Tpt1 / KptA family
AT2G45940	Chr02	18904413	18904738	+	1563	T	hypothetical protein (DUF295)
AT2G45950	Chr02	18907266	18907707	-	-9	P	SKP1-like 20
AT2G46320	Chr02	19015559	19015993	+	-32	P	Mitochondrial substrate carrier family protein

AT2G46450	Chr02	19065930	19066665	+	0	G	cyclic nucleotide-gated channel 12
AT2G46910	Chr02	19273036	19273270	+	872	T	Plastid-lipid associated protein PAP / fibrillin family protein
AT3G01070	Chr03	20456	20690	+	0	G	early nodulin-like protein 16
AT3G02210	Chr03	412589	412903	-	-873	P	COBRA-like protein 1 precursor
AT3G03420	Chr03	812811	813119	+	0	G	Ku70-binding family protein
AT3G05700	Chr03	1682072	1682306	-	0	G	Drought-responsive family protein
AT3G07710	Chr03	2464680	2464914	-	-24	P	hypothetical protein
AT3G08490	Chr03	2576604	2576838	-	0	G	delta-latroinsectotoxin-Lt1a protein
AT3G08690	Chr03	2641289	2641523	+	350	T	ubiquitin-conjugating enzyme 11
AT3G08730	Chr03	2654024	2654328	-	36	P	protein-serine kinase 1
AT3G08890	Chr03	2705275	2705509	+	-1142	T	hypothetical protein (Protein of unknown function, DUF538)
AT3G08990	Chr03	2743288	2743804	+	18	P	Yippee family putative zinc-binding protein
AT3G09400	Chr03	2892174	2892408	-	0	G	pol-like 3
AT3G11010	Chr03	3452551	3452785	-	0	G	receptor like protein 34
AT3G11370	Chr03	3559803	3560037	+	0	G	Cysteine/Histidine-rich C1 domain family protein
AT3G12290	Chr03	3920677	3920937	+	1344	T	Amino acid dehydrogenase family protein
AT3G13470	Chr03	4389627	4389861	+	0	G	TCP-1/cpn60 chaperonin family protein
AT3G13610	Chr03	4447026	4447484	+	0	G	2-oxoglutarate (2OG) and Fe(II)-dependent oxygenase superfamily protein
AT3G13770	Chr03	4519011	4519333	+	-459	P	Pentatricopeptide repeat (PPR) superfamily protein
AT3G13940	Chr03	4601639	4601890	-	0	G	DNA binding / DNA-directed RNA polymerase
AT3G14730	Chr03	4950571	4950805	-	0	G	Pentatricopeptide repeat (PPR) superfamily protein
AT3G14740	Chr03	4952341	4952676	-	0	G	RING/FYVE/PHD zinc finger superfamily protein
AT3G14890	Chr03	5009230	5009464	+	0	G	phosphoesterase
AT3G15380	Chr03	5194771	5195033	+	0	G	Plasma-membrane choline transporter family protein
AT3G16000	Chr03	5435727	5435961	-	0	G	MAR binding filament-like protein 1

AT3G16390	Chr03	5562462	5562696	+	21	P	nitrile specifier protein 3
AT3G17205	Chr03	5875885	5876143	+	0	G	ubiquitin protein ligase 6
AT3G17730	Chr03	6068399	6068633	+	0	G	NAC domain containing protein 57
AT3G03705	Chr03	6380533	6380884	-	616	T	NA
AT3G19670	Chr03	6837569	6837803	-	-181	P	pre-mRNA-processing protein 40B
AT3G20362	Chr03	7104249	7104493	+	554	P	hypothetical protein
AT3G20730	Chr03	7245339	7245688	+	0	G	PPR superfamily protein
AT3G21055	Chr03	7375758	7376094	-	1260	T	photosystem II subunit T
AT3G21473	Chr03	7564836	7565134	+	-181	P	hypothetical protein
AT3G23030	Chr03	8181590	8181854	-	0	G	indole-3-acetic acid inducible 2
AT3G23330	Chr03	8347746	8348075	+	746	T	Tetratricopeptide repeat (TPR)-like superfamily protein
AT3G23690	Chr03	8531032	8531347	-	169	T	basic helix-loop-helix (bHLH) DNA-binding superfamily protein
AT3G24715	Chr03	9026912	9027259	+	0	G	kinase superfamily with octicosapeptide/Phox/Bem1p domain-containing protein
AT3G25010	Chr03	9112586	9112923	-	-6	P	receptor like protein 41
AT3G25140	Chr03	9153250	9153484	+	0	G	Nucleotide-diphospho-sugar transferases superfamily protein
AT3G25540	Chr03	9275487	9275721	+	0	G	TRAM, LAG1 and CLN8 (TLC) lipid-sensing domain containing protein
AT3G26600	Chr03	9770272	9770730	+	0	G	armadillo repeat only 4
AT3G27180	Chr03	10029135	10029369	-	0	G	S-adenosyl-L-methionine-dependent methyltransferases superfamily protein
AT3G27870	Chr03	10328778	10329012	+	-1652	T	ATPase E1-E2 type family protein / haloacid dehalogenase-like hydrolase family protein
AT3G28291	Chr03	10550734	10551238	+	59	P	hypothetical protein
AT3G28480	Chr03	10677596	10677830	-	0	G	Oxoglutarate/iron-dependent oxygenase
AT3G28770	Chr03	10797629	10797874	+	0	G	transmembrane protein, putative (DUF1216)
AT3G28770	Chr03	10799537	10799771	+	0	G	transmembrane protein, putative (DUF1216)
AT3G28880	Chr03	10893468	10893731	+	-17	P	serine/threonine-protein phosphatase 6 regulatory ankyrin repeat subunit

AT3G06355	Chr03	14192942	14205144	+	-532	P	NA
AT3G45320	Chr03	16631710	16631950	+	195	T	transmembrane protein
AT3G46060	Chr03	16919184	16919418	+	0	G	RAB GTPase homolog 8A
AT3G46720	Chr03	17211685	17211919	-	0	G	UDP-Glycosyltransferase superfamily protein
AT3G47110	Chr03	17351278	17351577	-	-474	P	Leucine-rich repeat protein kinase family protein
AT3G47990	Chr03	17714443	17714677	-	0	G	SUGAR-INSENSITIVE 3
AT3G49340	Chr03	18293745	18293979	-	0	G	Cysteine proteinases superfamily protein
AT3G08075	Chr03	18440886	18441120	+	-665	P	NA
AT3G49740	Chr03	18446616	18446850	+	-992	P	Tetratricopeptide repeat (TPR)-like superfamily protein
AT3G49810	Chr03	18476594	18476859	-	0	G	ARM repeat superfamily protein
AT3G49845	Chr03	18485957	18486204	+	0	G	cysteine-rich TM module stress tolerance protein
AT3G49860	Chr03	18492778	18493012	-	-654	P	ADP-ribosylation factor-like A1B
AT3G50030	Chr03	18549413	18549647	-	0	G	ARM-repeat/Tetratricopeptide repeat (TPR)-like protein
AT3G51670	Chr03	19169085	19169402	+	0	G	SEC14 cytosolic factor family protein / phosphoglyceride transfer family protein
AT3G53270	Chr03	19752166	19752472	-	0	G	Small nuclear RNA activating complex (SNAPc), subunit SNAP43 protein
AT3G53310	Chr03	19766724	19767050	-	0	G	AP2/B3-like transcriptional factor family protein
AT3G56330	Chr03	20890993	20891227	+	875	T	N2,N2-dimethylguanosine tRNA methyltransferase
AT3G08775	Chr03	21062927	21063275	-	-327	P	NA
AT3G57080	Chr03	21125164	21125435	+	-426	P	Eukaryotic rpb5 RNA polymerase subunit family protein
AT3G57190	Chr03	21165508	21165765	+	-471	P	peptide chain release factor
AT3G57330	Chr03	21216307	21216614	-	0	G	autoinhibited Ca2+-ATPase 11
AT3G57390	Chr03	21233549	21233783	+	-50	P	AGAMOUS-like 18
AT3G57780	Chr03	21401284	21401518	-	0	G	nucleolar-like protein
AT3G57870	Chr03	21429838	21430161	-	399	T	sumo conjugation enzyme 1
AT3G57880	Chr03	21433416	21433650	-	0	G	Calcium-dependent lipid-binding (CaLB domain) plant phosphoribosyltransferase family protein

AT3G58100	Chr03	21515424	21515732	-	962	T	plasmodesmata callose-binding protein 5
AT3G58200	Chr03	21561336	21561570	-	63	P	TRAF-like family protein
AT3G59050	Chr03	21826644	21827048	-	0	G	polyamine oxidase 3
AT3G59420	Chr03	21962907	21963141	-	156	T	crinkly4
AT3G59550	Chr03	21997003	21997264	+	81	P	Rad21/Rec8-like family protein
AT3G59880	Chr03	22122011	22122245	+	1252	T	hypothetical protein
AT3G61415	Chr03	22726026	22726260	-	281	T	SKP1-like 21
AT3G62340	Chr03	23070509	23070743	-	581	T	WRKY family transcription factor
AT4G00180	Chr04	75148	75383	-	0	G	Plant-specific transcription factor YABBY family protein
AT4G00520	Chr04	227738	227972	+	161	P	Acyl-CoA thioesterase family protein
AT4G03855	Chr04	470223	470457	+	942	T	NA
AT4G01180	Chr04	503418	503684	-	-157	P	XH/XS domain-containing protein
AT4G01430	Chr04	586046	586318	+	0	G	nodulin MtN21 /EamA-like transporter family protein
AT4G01850	Chr04	798126	798360	-	37	P	S-adenosylmethionine synthetase 2
AT4G01915	Chr04	827960	828323	+	904	T	hypothetical protein
AT4G04075	Chr04	973405	973761	-	-31	P	NA
AT4G02655	Chr04	1162581	1162845	+	0	G	transmembrane protein
AT4G05015	Chr04	2460564	2460798	-	-61	P	NA
AT4G08950	Chr04	5740417	5740981	+	0	G	Phosphate-responsive 1 family protein
AT4G09640	Chr04	6090654	6090904	-	0	G	magnesium transporter, putative (DUF803)
AT4G10260	Chr04	6372235	6372682	-	331	T	pfkB-like carbohydrate kinase family protein
AT4G10520	Chr04	6502166	6502529	+	0	G	Subtilase family protein
AT4G10600	Chr04	6548028	6548361	-	0	G	RING/FYVE/PHD zinc finger superfamily protein
AT4G11550	Chr04	7001085	7001493	-	0	G	Cysteine/Histidine-rich C1 domain family protein
AT4G11780	Chr04	7087758	7088159	+	0	G	GAR2-like protein
AT4G11830	Chr04	7119407	7119646	-	0	G	phospholipase D gamma 2
AT4G13530	Chr04	7867157	7867535	+	0	G	transmembrane protein
AT4G13650	Chr04	7941977	7942310	-	0	G	Pentatricopeptide repeat (PPR) superfamily protein
AT4G14300	Chr04	8231099	8231333	+	214	P	RNA-binding (RRM/RBD/RNP motifs) family protein
AT4G06210	Chr04	8423058	8423427	-	0	G	NA

AT4G16440	Chr04	9272178	9272646	-	-743	P	ferredoxin hydrogenase
AT4G18950	Chr04	10375739	10375973	+	0	G	Integrin-linked protein kinase family
AT4G19660	Chr04	10697501	10697772	-	0	G	NPR1-like protein 4
AT4G19900	Chr04	10790468	10790722	-	0	G	alpha 1,4-glycosyltransferase family protein
AT4G20790	Chr04	11136089	11136624	-	67	P	Leucine-rich repeat protein kinase family protein
AT4G21070	Chr04	11246954	11247314	+	-857	P	breast cancer susceptibility1
AT4G21300	Chr04	11336929	11337298	+	0	G	Tetratricopeptide repeat (TPR)-like superfamily protein
AT4G21700	Chr04	11530804	11531084	+	0	G	DUF2921 family protein, putative (DUF2921)
AT4G21820	Chr04	11577830	11578160	+	471	P	binding / calmodulin binding protein
AT4G22130	Chr04	11723552	11723786	+	0	G	STRUBBELIG-receptor family 8
AT4G26555	Chr04	13406050	13406284	-	-58	P	FKBP-like peptidyl-prolyl cis-trans isomerase family protein
AT4G27020	Chr04	13570496	13570730	-	0	G	inositol-1,4,5-trisphosphate 5-phosphatase
AT4G29010	Chr04	14298525	14298759	-	0	G	Enoyl-CoA hydratase/isomerase family
AT4G30470	Chr04	14894391	14894844	+	0	G	NAD(P)-binding Rossmann-fold superfamily protein
AT4G30760	Chr04	14983086	14983347	+	766	P	Putative endonuclease or glycosyl hydrolase
AT4G08955	Chr04	16386944	16387178	+	0	G	NA
AT4G34760	Chr04	16582739	16582973	-	0	G	SAUR-like auxin-responsive protein family
AT4G34890	Chr04	16622640	16622874	-	0	G	xanthine dehydrogenase 1
AT4G35500	Chr04	16858547	16858844	+	0	G	Protein kinase superfamily protein
AT4G37870	Chr04	17805208	17805442	-	0	G	phosphoenolpyruvate carboxykinase 1
AT4G38020	Chr04	17861699	17861933	+	381	P	tRNA/rRNA methyltransferase (SpoU) family protein
AT4G39330	Chr04	18292094	18292543	+	1100	T	cinnamyl alcohol dehydrogenase 9
AT5G01440	Chr05	180455	180689	+	0	G	hypothetical protein
AT5G02140	Chr05	423058	423330	+	-8	P	Pathogenesis-related thaumatin superfamily protein

AT5G03730	Chr05	977555	977830	-	0	G	Protein kinase superfamily protein
AT5G04540	Chr05	1301547	1301781	-	0	G	Myotubularin-like phosphatases II superfamily
AT5G05560	Chr05	1641062	1641296	+	-186	P	E3 ubiquitin ligase
AT5G05820	Chr05	1753252	1753511	-	0	G	Nucleotide-sugar transporter family protein
AT5G01255	Chr05	1930917	1931331	+	-168	P	NA
AT5G06560	Chr05	2004968	2005202	-	0	G	myosin-binding protein (Protein of unknown function, DUF593)
AT5G07590	Chr05	2402659	2402893	-	0	G	Transducin/WD40 repeat-like superfamily protein
AT5G08630	Chr05	2802653	2803039	-	325	P	DDT domain-containing protein
AT5G09690	Chr05	3002532	3002766	-	0	G	magnesium transporter 7
AT5G10040	Chr05	3143725	3143999	+	139	P	transmembrane protein
AT5G10100	Chr05	3157973	3158207	+	0	G	Haloacid dehalogenase-like hydrolase (HAD) superfamily protein
AT5G10240	Chr05	3215840	3216074	-	509	T	asparagine synthetase 3
AT5G10290	Chr05	3235460	3235796	-	0	G	leucine-rich repeat transmembrane protein kinase family protein
AT5G10340	Chr05	3253062	3253361	+	89	P	F-box family protein
AT5G13200	Chr05	4208437	4208710	+	1789	T	GRAM domain family protein
AT5G13680	Chr05	4414711	4414962	-	786	P	IKI3 family protein
AT5G13820	Chr05	4461474	4461708	+	37	P	telomeric DNA binding protein 1
AT5G14320	Chr05	4618337	4618571	-	458	T	Ribosomal protein S13/S18 family
AT5G15390	Chr05	4993366	4993600	+	0	G	tRNA/rRNA methyltransferase (SpoU) family protein
AT5G16170	Chr05	5277748	5278134	+	0	G	Core-2/I-branching beta-1,6-N-acetylglucosaminyltransferase family protein
AT5G17330	Chr05	5710992	5711246	+	0	G	glutamate decarboxylase
AT5G17430	Chr05	5744526	5744760	-	0	G	Integrase-type DNA-binding superfamily protein
AT5G02745	Chr05	5795032	5795266	-	47	P	NA
AT5G17850	Chr05	5899723	5899968	+	0	G	Sodium/calcium exchanger family protein
AT5G18310	Chr05	6062254	6062574	+	0	G	ubiquitin hydrolase
AT5G18500	Chr05	6138572	6138948	+	43	P	Protein kinase superfamily protein
AT5G19190	Chr05	6457523	6457939	+	0	G	hypothetical protein

AT5G19315	Chr05	6505321	6505584	+	921	T	Defensin-like (DEFL) family protein
AT5G20010	Chr05	6758500	6758734	+	0	G	RAS-related nuclear protein-1
AT5G20510	Chr05	6942545	6942803	-	234	T	alfin-like 5
AT5G22010	Chr05	7287800	7288104	-	-518	P	replication factor C1
AT5G22720	Chr05	7551463	7551697	-	-406	P	F-box/FBD/LRR protein
AT5G23040	Chr05	7730815	7731049	-	123	T	cell growth defect factor-like protein (DUF3353)
AT5G24760	Chr05	8496288	8496528	-	0	G	GroES-like zinc-binding dehydrogenase family protein
AT5G25230	Chr05	8740945	8741224	+	0	G	Ribosomal protein S5/Elongation factor G/III/V family protein
AT5G25280	Chr05	8774020	8774517	+	0	G	serine-rich protein-like protein
AT5G04055	Chr05	8958329	8958563	+	-908	P	NA
AT5G26210	Chr05	9159513	9159810	-	0	G	alfin-like 4
AT5G26570	Chr05	9262130	9262364	+	0	G	chloroplastidic phosphoglucan, water dikinase (ATGWD3)
AT5G27410	Chr05	9679593	9679827	+	-31	P	D-aminoacid aminotransferase-like PLP-dependent enzymes superfamily protein
AT5G28050	Chr05	10044793	10045037	-	0	G	Cytidine/deoxycytidylate deaminase family protein
AT5G28180	Chr05	10162808	10163048	+	0	G	Galactose oxidase/kelch repeat superfamily protein
AT5G28460	Chr05	10376046	10376293	+	0	G	Pentatricopeptide repeat (PPR) superfamily protein
AT5G35620	Chr05	13825606	13825864	-	0	G	Eukaryotic initiation factor 4E protein
AT5G35840	Chr05	14009845	14010079	+	0	G	phytochrome C
AT5G37430	Chr05	14841625	14842020	+	-608	P	hypothetical protein (DUF577)
AT5G37570	Chr05	14925696	14925930	-	0	G	Pentatricopeptide repeat (PPR-like) superfamily protein
AT5G38200	Chr05	15257931	15258326	+	0	G	Class I glutamine amidotransferase-like superfamily protein
AT5G39000	Chr05	15611479	15611739	+	-203	P	Malectin/receptor-like protein kinase family protein
AT5G39510	Chr05	15821397	15821631	+	221	T	Vesicle transport v-SNARE family protein
AT5G40410	Chr05	16171406	16171644	+	151	T	Tetratricopeptide repeat (TPR)-like superfamily protein
AT5G40440	Chr05	16187581	16187815	+	0	G	mitogen-activated protein

							kinase kinase 3
AT5G40450	Chr05	16188939	16189173	-	0	G	A-kinase anchor-like protein
AT5G41110	Chr05	16451766	16452000	+	413	P	meiosis chromosome segregation family protein
AT5G41290	Chr05	16512429	16512748	+	0	G	Receptor-like protein kinase-related family protein
AT5G42050	Chr05	16815679	16816532	+	0	G	DCD (Development and Cell Death) domain protein
AT5G42325	Chr05	16924045	16924332	-	663	T	Transcription factor IIS protein
AT5G42470	Chr05	16982727	16982961	+	0	G	BRCA1-A complex subunit BRE-like protein
AT5G06395	Chr05	17169165	17169441	+	-1002	T	NA
AT5G42830	Chr05	17176321	17176555	+	0	G	HXXXD-type acyl-transferase family protein
AT5G43200	Chr05	17346227	17346674	-	379	P	Zinc finger, C3HC4 type (RING finger) family protein
AT5G44770	Chr05	18065545	18065784	+	0	G	Cysteine/Histidine-rich C1 domain family protein
AT5G44790	Chr05	18078909	18079143	-	0	G	copper-exporting ATPase / responsive-to-antagonist 1 / copper-transporting ATPase (RAN1)
AT5G45340	Chr05	18369676	18369914	-	0	G	cytochrome P450, family 707, subfamily A, polypeptide 3
AT5G45430	Chr05	18407401	18407677	+	151	P	Protein kinase superfamily protein
AT5G45520	Chr05	18454401	18454810	-	-922	P	Leucine-rich repeat (LRR) family protein
AT5G06885	Chr05	19047511	19047745	-	-709	P	NA
AT5G49580	Chr05	20125773	20126036	-	0	G	Chaperone DnaJ-domain superfamily protein
AT5G50240	Chr05	20451795	20452090	+	409	P	protein-l-isoaspartate methyltransferase 2
AT5G50290	Chr05	20461113	20461366	+	-538	P	wall-associated receptor kinase galacturonan-binding protein
AT5G50530	Chr05	20573389	20573792	-	1660	T	CBS / octicosapeptide/Phox/Bemp1 (PB1) domains-containing protein
AT5G50530	Chr05	20574344	20574746	-	705	T	CBS / octicosapeptide/Phox/Bemp1 (PB1) domains-containing protein
AT5G50550	Chr05	20577689	20577934	+	0	G	Transducin/WD40 repeat-like

							superfamily protein
AT5G50570	Chr05	20583984	20584218	-	0	G	Squamosa promoter-binding protein-like (SBP domain) transcription factor family protein
AT5G50580	Chr05	20586140	20586431	+	0	G	SUMO-activating enzyme 1B
AT5G51190	Chr05	20800894	20801159	-	0	G	Integrase-type DNA-binding superfamily protein
AT5G51450	Chr05	20894628	20894862	+	0	G	RPM1 interacting protein 3
AT5G52660	Chr05	21360389	21360713	-	0	G	Homeodomain-like superfamily protein
AT5G53020	Chr05	21500044	21500306	+	0	G	Ribonuclease P protein subunit P38-like protein
AT5G54035	Chr05	21926328	21926631	+	240	T	hypothetical protein
AT5G54080	Chr05	21944828	21945096	+	-903	P	homogentisate 1,2-dioxygenase
AT5G54750	Chr05	22241539	22242022	+	299	P	Transport protein particle (TRAPP) component
AT5G55660	Chr05	22538810	22539044	+	0	G	DEK domain-containing chromatin associated protein
AT5G56150	Chr05	22729312	22729584	+	266	T	ubiquitin-conjugating enzyme 30
AT5G56350	Chr05	22824065	22824356	-	0	G	Pyruvate kinase family protein
AT5G56560	Chr05	22900440	22900724	+	0	G	FBD, F-box and Leucine Rich Repeat domains containing protein
AT5G57480	Chr05	23279775	23280009	-	0	G	P-loop containing nucleoside triphosphate hydrolases superfamily protein
AT5G57560	Chr05	23307402	23307846	-	0	G	Xyloglucan endotransglucosylase/hydrolase family protein
AT5G59750	Chr05	24074707	24075004	+	1560	T	monofunctional riboflavin biosynthesis protein RIBA 3
AT5G60070	Chr05	24193667	24193901	-	-890	P	ankyrin repeat family protein
AT5G60150	Chr05	24218439	24218737	+	606	P	hypothetical protein
AT5G61260	Chr05	24637332	24637566	+	0	G	Plant calmodulin-binding protein-like protein
AT5G62050	Chr05	24927080	24927323	-	113	P	inner membrane OXA1-like protein
AT5G62890	Chr05	25243337	25243571	+	37	P	Xanthine/uracil permease family protein
AT5G63130	Chr05	25323860	25324180	+	1045	T	Octicosapeptide/Phox/Bem1p family protein
AT5G63180	Chr05	25342095	25342329	-	0	G	Pectin lyase-like superfamily

							protein
AT5G64030	Chr05	25624290	25624524	+	0	G	S-adenosyl-L-methionine-dependent methyltransferases superfamily protein
AT5G66840	Chr05	26693232	26693567	-	1954	T	SAP domain-containing protein
ATMG00010		572	835	-	31	P	NA
ATMG00080		24889	25434	-	454	P	NA
ATMG00110		30730	30995	+	0	G	NA
ATMG00140		34466	34721	+	-6	P	NA
ATMG00200		56413	56705	-	-469	P	hypothetical protein
ATMG00220		60454	60775	+	0	G	cytochrome b
ATMG00260		75769	76164	+	-166	P	NA
ATMG00270		77229	77672	-	-191	P	NA
ATMG00310		91993	92589	-	-944	P	uncharacterized mitochondrial protein AtMg00310-like
ATMG00370		105719	106332	-	-472	P	uncharacterized mitochondrial protein AtMg00370-like
ATMG00430		114864	116421	+	211	P	NA
ATMG00440		117166	117421	+	997	T	NA
ATMG00450		118411	118645	-	1173	T	NA
ATMG00480		129624	129902	+	-146	P	Plant mitochondrial ATPase, F0 complex, subunit 8 protein
ATMG00513		141677	142018	-	0	G	NA
ATMG00590		171287	171521	+	1608	T	Cytochrome b/b6 protein
ATMG00630		182382	183132	+	-354	P	NA
ATMG00630		183588	183836	+	601	T	NA
ATMG00710		206383	206740	-	1354	T	uncharacterized mitochondrial protein AtMg00710-like
ATMG00710		207966	208362	-	-249	P	uncharacterized mitochondrial protein AtMg00710-like
ATMG00720		208533	209360	+	-553	P	NA
ATMG00770		222569	223217	-	96	P	NA
ATMG00820		229052	229291	-	-86	P	uncharacterized mitochondrial protein AtMg00820-like
ATMG00870		237869	238114	-	-510	P	NA
ATMG00940		250892	251410	-	746	T	NA
ATMG00960		257155	257402	-	198	T	Cytochrome C assembly protein
ATMG01030		263334	263664	-	934	T	uncharacterized mitochondrial protein AtMg01030
ATMG01030		264270	264626	-	-15	P	NA
ATMG01040		270735	271132	-	-372	P	NA
ATMG01170		297442	298542	-	0	G	NA
ATMG01190		302717	303021	-	820	T	ATPase, F1 complex, alpha subunit protein

ATMG01200	304680	305019	-	-129	P	NA
ATMG01220	305374	305763	-	-6	P	hypothetical protein
ATMG01260	312504	312790	-	0	G	NA
ATMG01360	350912	351186	-	0	G	NA
ATMG01360	352015	352357	-	-773	P	NA
ATMG01400	362701	362966	+	-892	P	NA
ATMG01400	364804	365187	+	1270	T	NA
ATCG00040	4	7991	-	-361	P	NA
ATCG00180	8262	36249	-	829	T	NA
ATCG00790	36401	128449	-	227	T	NA
ATCG01240	139716	141331	+	-181	P	NA
ATCG01250	141475	143998	+	0	G	NA
ATCG01280	145237	154208	-	0	G	NA
AT1G58150	21527527	21528164	+	-2258	I	
AT1G52200	19448761	19448995	-	-5247	I	
AT1G41830	15599248	15599667	-	8509	I	
AT1G40390	15370548	15370782	-	64009	I	
AT1G40129	15272453	15272869	+	32108	I	
AT1G40104	15143202	15143436	+	61367	I	
AT1G40104	15100175	15100534	+	18402	I	
AT1G39070	14721755	14722090	+	-11603	I	
AT1G23010	8149128	8149362	+	2127	I	
AT1G38790	14592359	14592636	-	-47969	I	
AT1G79250	29809037	29809347	-	3087	I	
AT1G12420	4233779	4234023	-	-4690	I	
AT1G61860	22861827	22862119	-	2925	I	
AT1G63750	23656667	23656930	+	5950	I	
AT1G58340	21656062	21656296	+	3191	I	
AT1G11270	3784688	3784954	-	2167	I	
AT1G76790	28821122	28821412	-	2460	I	
AT2G01021	7736	8084	+	1339	I	
AT2G4816	19697763	19698067	-	-1094	I	
AT2G05865	5404050	5404357	+	-7525	I	
AT2G12646	5200395	5200629	-	-31209	I	
AT2G12646	5202081	5202591	-	-33033	I	
AT2G12646	5203090	5203818	-	-34151	I	
AT2G09795	3662698	3662987	-	9386	I	
AT2G09840	3715410	3715648	-	-9935	I	
AT2G09840	3718025	3718342	-	-12589	I	
AT2G12646	5198819	5200247	-	-30230	I	
AT2G07741	3504932	3505219	+	2756	I	
AT2G05395	3506250	3506983	+	-3074	I	
AT2G07981	3605710	3607434	+	-8414	I	
AT2G07981	3607585	3607921	+	-7233	I	

AT2G07732	3470285	3470637	-	-1562	I
AT2G07718	3418083	3418781	+	-6253	I
AT2G07721	3436194	3436840	-	-1238	I
AT2G07701	3369365	3369739	+	-2280	I
AT2G07696	3354007	3354366	-	-1958	I
AT2G07678	3279172	3279575	+	-1724	I
AT2G07565	3185558	3185951	+	-1314	I
AT2G04745	1435912	1436146	-	2749	I
AT2G08986	3626749	3627537	+	9085	I
AT2G35075	14788602	14788936	+	2309	I
AT2G05215	3331903	3332558	+	-2180	I
AT2G29605	12662061	12662322	-	-2104	I
AT2G05230	1906063	1906297	-	-3355	I
AT3G28270	10536531	10536765	+	-1357	I
AT3G61320	22696706	22697575	+	3267	I
AT3G09245	22060855	22061263	+	1883	I
AT3G50300	18642523	18642831	+	-1183	I
AT3G05635	10965955	10966324	-	-1423	I
AT3G29255	11205422	11205668	+	-4041	I
AT3G31910	12915474	12915708	-	-5200	I
AT3G32980	13587521	13587811	-	-57568	I
AT3G32980	13588844	13592548	-	-60598	I
AT3G61340	22700170	22700479	-	2157	I
AT3G41761	14191725	14192517	+	-3796	I
AT4G14615	8382425	8382871	+	-1079	I
AT4G06701	4000422	4000656	-	-59742	I
AT4G06701	4000980	4001250	-	-60318	I
AT4G06701	4005588	4005991	-	-64992	I
AT4G06744	4007230	4007524	-	65093	I
AT4G06744	4009586	4010302	-	62526	I
AT4G06744	4010712	4011179	-	61525	I
AT4G06701	3976491	3976815	-	-35856	I
AT4G06701	3977499	3978237	-	-37071	I
AT4G06701	3978616	3979033	-	-38027	I
AT4G06701	3979199	3979633	-	-38619	I
AT4G06701	3979962	3980411	-	-39389	I
AT4G06701	3982357	3982720	-	-41741	I
AT4G06701	3984280	3984776	-	-43731	I
AT4G06701	3985296	3985654	-	-44678	I
AT4G06701	3985921	3986235	-	-45281	I
AT4G06701	3987355	3987616	-	-46688	I
AT4G06526	3259254	3259488	-	61574	I
AT4G06701	3950527	3956000	-	-12466	I
AT4G06701	3965732	3966021	-	-25079	I

AT4G06701	3966429	3966766	-	-25800	I
AT4G06701	3967100	3967466	-	-26486	I
AT4G06701	3969155	3969692	-	-28626	I
AT4G06701	3972616	3972856	-	-31939	I
AT4G06701	3974095	3974329	-	-33415	I
AT4G06701	3974672	3975020	-	-34049	I
AT4G06701	3975707	3976100	-	-35106	I
AT4G05612	2951191	2951425	+	-7786	I
AT4G06479	3054548	3054821	+	-12819	I
AT4G06526	3221806	3222123	-	98981	I
AT4G06526	3249435	3249669	-	71393	I
AT4G06526	3252250	3252531	-	68555	I
AT4G03420	1509978	1510215	+	-1501	I
AT4G32600	15725149	15725769	+	2102	I
AT4G38620	18056304	18056567	+	3075	I
AT5G32440	11860596	11861233	+	-215803	I
AT5G32440	11876468	11876713	+	-200127	I
AT5G32440	11957589	11957838	+	-119004	I
AT5G32440	11958656	11959243	+	-117768	I
AT5G32440	11963598	11963843	+	-112997	I
AT5G32440	12046645	12046880	+	-29955	I
AT5G32440	12064485	12064800	+	-12075	I
AT5G30520	11708782	11709241	-	-72373	I
AT5G30520	11710147	11710396	-	-73633	I
AT5G30520	11723379	11723869	-	-86986	I
AT5G30520	11724119	11724593	-	-87718	I
AT5G30520	11726189	11729943	-	-91428	I
AT5G30520	11730334	11733991	-	-95524	I
AT5G30520	11734278	11735476	-	-98239	I
AT5G30520	11735744	11736294	-	-99381	I
AT5G30520	11754395	11754629	-	-117874	I
AT5G30520	11795206	11795440	-	-158685	I
AT5G29560	11184912	11185260	-	6881	I
AT5G29560	11185468	11186674	-	5896	I
AT5G30520	11701933	11703669	-	-66163	I
AT5G30520	11703869	11704380	-	-67486	I
AT5G30520	11704526	11704812	-	-68031	I
AT5G30520	11705176	11705645	-	-68772	I
AT5G30520	11706835	11707170	-	-70364	I
AT5G30520	11707396	11707683	-	-70901	I
AT5G30520	11708076	11708494	-	-71647	I
AT5G13200	4210099	4210338	+	3434	I
AT5G13210	4211409	4211836	+	-2333	I
AT5G61020	24557277	24557524	-	2479	I

AT5G13210	4212667	4213033	+	-1105	I
ATMG01370	357587	358263	-	3127	I
ATMG01290	322009	322626	+	-1942	I
ATMG01320	339478	339855	-	-6561	I
ATMG01350	344845	345108	+	-1781	I
ATMG01180	299663	300250	-	1345	I
ATMG01060	276603	276839	+	1822	I
ATMG01040	269074	269357	-	1346	I
ATMG00630	185379	185831	+	2494	I
ATMG00580	159882	160180	+	-1662	I
ATMG00470	123681	124114	-	3946	I
ATMG00400	109706	110142	-	1479	I
ATMG00290	84704	85077	-	-1774	I
ATMG00220	63012	63279	+	2910	I
ATMG00240	66260	66533	-	2857	I
ATMG00240	67458	68133	-	1458	I
ATMG00260	73386	73844	+	-2517	I
ATMG00170	45814	46704	+	-1854	I
ATMG01360	349080	349585	-	2081	I
ATCG01130	130977	131211	-	-1850	I
ATCG01130	132273	132882	-	-3333	I
ATCG01130	133540	133924	-	-4488	I
ATCG01230	134179	135750	+	-4892	I

Footnote: G: gene; P: promoter; T: terminator; I: intergenic; Start: peak start location on the genome; End: peak end location on the genome
